# Endocrine Therapy Synergizes with SMAC Mimetics to Potentiate Antigen Presentation and Tumor Regression in Hormone Receptor–Positive Breast Cancer

**DOI:** 10.1158/0008-5472.CAN-23-1711

**Published:** 2023-07-14

**Authors:** Francisco Hermida-Prado, Yingtian Xie, Shira Sherman, Zsuzsanna Nagy, Douglas Russo, Tara Akhshi, Zhengtao Chu, Avery Feit, Marco Campisi, Minyue Chen, Agostina Nardone, Cristina Guarducci, Klothilda Lim, Alba Font-Tello, Irene Lee, Juana García-Pedrero, Israel Cañadas, Judith Agudo, Ying Huang, Tal Sella, Qingchun Jin, Nabihah Tayob, Elizabeth A. Mittendorf, Sara M. Tolaney, Xintao Qiu, Henry Long, William F. Symmans, Jia-Ren Lin, Sandro Santagata, Isabelle Bedrosian, Denise A. Yardley, Ingrid A. Mayer, Edward T. Richardson, Giacomo Oliveira, Catherine J. Wu, Eugene F. Schuster, Mitch Dowsett, Alana L. Welm, David Barbie, Otto Metzger, Rinath Jeselsohn

**Affiliations:** 1 Center for Functional Cancer Epigenetics, Dana-Farber Cancer Institute, Boston, Massachusetts.; 2 Department of Medical Oncology, Dana-Farber Cancer Institute, Boston, Massachusetts.; 3 University of Oviedo, Instituto de Investigación Sanitaria del Principado de Asturias (ISPA), IUOPA, Oviedo, Spain.; 4 CIBERONC, Instituto de Salud Carlos III, Madrid, Spain.; 5 Department of Data Science, Dana-Farber Cancer Institute, Boston, Massachusetts.; 6 Huntsman Cancer Institute, Department of Oncological Sciences, University of Utah, Salt Lake City, Utah.; 7 Department of Immunology, Harvard Medical School, Boston, Massachusetts.; 8 Blood Cell Development and Function Program, Fox Chase Cancer Center, Philadelphia, Pennsylvania.; 9 Harvard Medical School, Boston, Massachusetts.; 10 Department of Cancer Immunology and Virology, Dana-Farber Cancer Institute, Boston, Massachusetts.; 11 Department of Oncologic Pathology, Dana-Farber Cancer Institute, Boston, Massachusetts.; 12 Breast Oncology Program, Dana-Farber Brigham Cancer Center, Boston, Massachusetts.; 13 Division of Breast Surgery, Department of Surgery, Brigham and Women's Hospital, Boston, Massachusetts.; 14 Department of Pathology, MD Anderson Cancer Center, Houston, Texas.; 15 Ludwig Center at Harvard and Laboratory of Systems Pharmacology, Harvard Medical School, Boston, Massachusetts.; 16 Department of Systems Biology, Harvard Medical School, Boston, Massachusetts.; 17 Department of Pathology, Brigham and Women's Hospital, Boston, Massachusetts.; 18 Department of Breast Surgical Oncology, Division of Surgery, MD Anderson Cancer Center, Houston, Texas.; 19 Department of Medical Oncology, Sarah Cannon Cancer Center, Nashville, Tennessee.; 20 Tennessee Oncology, Nashville, Tennessee.; 21 Vanderbilt-Ingram Cancer Center, Vanderbilt University, Nashville, Tennessee.; 22 Broad Institute of MIT and Harvard, Cambridge, Massachusetts.; 23 Department of Medicine, Brigham and Women's Hospital, Boston, Massachusetts.; 24 The BC Now Toby Robins Research Centre at the Institute of Cancer Research, London, United Kingdom.; 25 Ralph Lauren Centre for BC Research, Royal Marsden Hospital, London, United Kingdom.; 26 The Royal Marsden Hospital, London, United Kingdom.

## Abstract

Immunotherapies have yet to demonstrate significant efficacy in the treatment of hormone receptor–positive (HR^+^) breast cancer. Given that endocrine therapy (ET) is the primary approach for treating HR^+^ breast cancer, we investigated the effects of ET on the tumor immune microenvironment (TME) in HR^+^ breast cancer. Spatial proteomics of primary HR^+^ breast cancer samples obtained at baseline and after ET from patients enrolled in a neoadjuvant clinical trial (NCT02764541) indicated that ET upregulated β2-microglobulin and influenced the TME in a manner that promotes enhanced immunogenicity. To gain a deeper understanding of the underlying mechanisms, the intrinsic effects of ET on cancer cells were explored, which revealed that ET plays a crucial role in facilitating the chromatin binding of RelA, a key component of the NF-κB complex. Consequently, heightened NF-κB signaling enhanced the response to interferon-gamma, leading to the upregulation of β2-microglobulin and other antigen presentation-related genes. Further, modulation of NF-κB signaling using a SMAC mimetic in conjunction with ET augmented T-cell migration and enhanced MHC-I-specific T-cell–mediated cytotoxicity. Remarkably, the combination of ET and SMAC mimetics, which also blocks prosurvival effects of NF-κB signaling through the degradation of inhibitors of apoptosis proteins, elicited tumor regression through cell autonomous mechanisms, providing additional support for their combined use in HR^+^ breast cancer.

**Significance:**

Adding SMAC mimetics to endocrine therapy enhances tumor regression in a cell autonomous manner while increasing tumor immunogenicity, indicating that this combination could be an effective treatment for HR^+^ patients with breast cancer.

## Graphical Abstract

**Figure:**
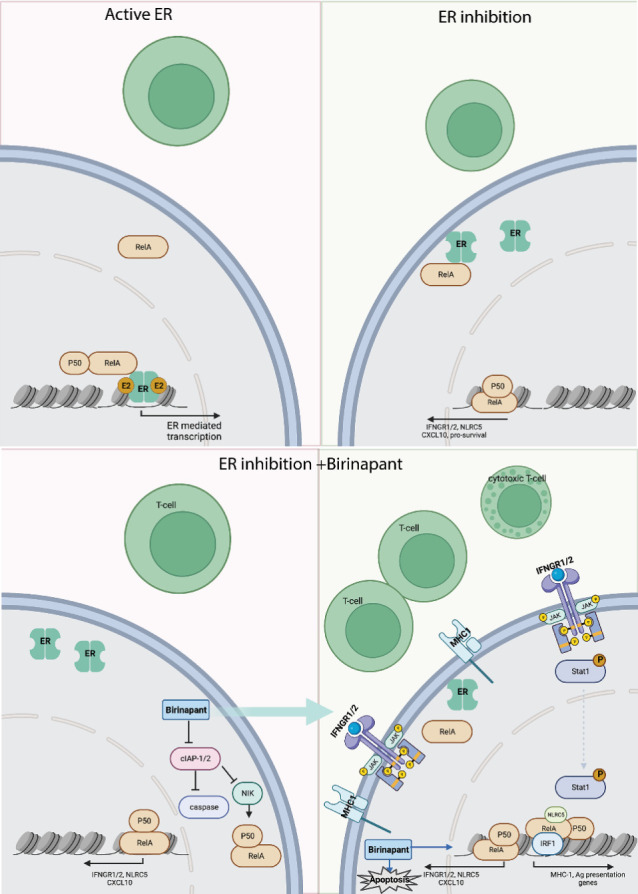

## Introduction

Immune-checkpoint inhibitors (ICI) and other immune therapies revolutionized the treatment paradigms of several malignancies (1). However, the use of ICIs has been limited in breast cancer and is mostly studied in the triple-negative breast cancer (TNBC) subtype (2, 3). ICIs have shown marginal efficacy in HR^+^ breast cancer, and there are no approved immunotherapy regimens in this breast cancer subtype. Nonetheless, several studies demonstrated a signal of clinical benefit in a limited number of patients (4–6), highlighting the potential utility of ICIs and unmet need to identify strategies to harness the immune system for the treatment of a broader population of patients with HR^+^ breast cancer.

The inherent resistance to ICIs in HR^+^ breast cancer is attributed to the low tumor mutational burden, low percentage of stromal tumor-infiltrating lymphocytes (sTIL), and low PD-L1 expression (7–9). Antigen presentation is required for the activation of cytotoxic CD8^+^ TILs and inadequate antigen presentation is another mechanism of tumor immune escape and resistance to ICIs (10). The major histocompatibility complex class I (MHC-I) is a key component of the antigen presentation machinery, responsible for the presentation of intracellular peptide antigens to the cell surface for specific cytotoxic T cell recognition. High MHC-I expression correlates with better responses to ICIs in lung cancer and melanoma (10, 11). The MHC-I complex is a heterodimer consisting of two polypeptide chains including α and β2-microglobulin (B2M). The α chain is polymorphic and encoded by an HLA gene (*HLA-A/B/C*), whereas the B2M subunit, encoded by the *B2M* gene, is not polymorphic. In cancers, MHC-I expression is mainly induced by the type II interferon, IFNγ, which is secreted primarily by T cells and natural killer (NK) cells and signals through the JAK–STAT pathway. A link between estrogen receptor α (ER) signaling and antigen presentation was demonstrated in a study that showed an inverse correlation between MHC-I and ER expression in primary breast cancer and normal breast tissue (12). Moreover, in metastatic HR^+^ breast cancer, higher ER signaling was associated with reduced antigen presentation and ICI resistance (13). Thus, there is evidence for the role of ER in modulating antigen presentation and sensitivity to ICIs; however, the underlying mechanisms of these effects remain elusive.

Previous studies showed that estradiol (E2) affects innate immune signaling pathways and myeloid cell development (14, 15). Additionally, several studies investigated the effects of ER signaling on the TME in tumors that are not ER dependent (16, 17). In this study, we sought to investigate the effects of ER blockade on the TME and tumor cell response to immune stimuli and antigen presentation in ER^+^ breast cancer with the aim to identify strategies to increase the immunogenicity of HR^+^ breast cancer.

## Materials and Methods

### Clinical trial design, tumor biopsies, and compliance with ethical standards

Palbociclib and ET for Lobular Breast Cancer Preoperative Study (PELOPS) was a multicenter, randomized, open-label phase II neoadjuvant clinical trial that enrolled patients with resectable early-stage, treatment-naïve hormone receptor–positive, HER2-negative breast cancer. The study included two parts and two patient cohorts based on the menopausal status (postmenopausal and premenopausal). The first part was a window-of-opportunity study in which the postmenopausal patients were randomized 1:1 to two weeks of treatment with tamoxifen (20 mg) versus letrozole (2.5 mg). In the second part of the study, the treatment part, the postmenopausal patient cohort rerandomized 2:1 to palbociclib plus letrozole versus letrozole alone. The premenopausal patients were randomized 2:1 to tamoxifen with lupron and palbociclib versus tamoxifen and lupron. Randomization was stratified by histologic subtype (IDC and ILC, mixed pathology was categorized as IDC), lymph node status, and tumor size. The main eligibility criteria included: Stage I to III histologically confirmed invasive carcinoma of the breast. A minimum tumor size of at least 1.5 cm determined by physical exam or imaging (based on the larger measurement), histologically confirmed hormone receptor–positive (ER and/or PR), HER2-negative. Cutoff values for positive/negative staining were in accordance with current ASCO/CAP (American Society of Clinical Oncology/College of American Pathologists) guidelines. Participants underwent a research biopsy at baseline and day 15. Core biopsies were obtained for snap-frozen samples and formalin-fixed paraffin-embedded (FFPE) blocks. FFPE tissue blocks, and/or cut slides were collected from the surgical excision at the end of the study. The clinicopathologic characteristics of the 111 patients in this analysis were comparable to the entire study population, and the clinical characteristics were well balanced between the treatment arms. The study was conducted in accordance with the International Conference on Harmonization Good Clinical Practice Standards and the Declaration of Helsinki. Institutional Review Board (IRB) approval was obtained at Dana-Farber/Harvard Cancer Center (DF/HCC; Dana-Farber/Harvard Cancer Center Protocol 16-052). The study was registered in ClinicalTrials.gov (NCT02764541). The DF/HCC Data and Safety Monitoring Committee, which is composed of clinical specialists with experience in oncology and who had no direct relationship with the study, reviewed and monitored toxicity and accrual data from the study. Participants provided written informed consent prior to the performance of any protocol-specific procedures or assessments. The study was an investigator-initiated trial funded by Pfizer. Palbociclib was supplied by the manufacturer (Pfizer). The funder had no role in data collection, data analysis, or data interpretation. The clinical primary end points of the study were: two primary end points: (1) window-of-opportunity part: compare the changes in Ki67 (fold change of Ki67 on log scale) between tamoxifen treatment and letrozole. (ii) treatment part: evaluate the effect of including palbociclib with ET compared with ET alone on pathologic response among all patients (based on RCB index). These results will be reported in a separate publication.

### Digital spatial profiling data generation

Digital spatial profiling (DSP; NanoString) was performed as described previously (18). Tissue slides were stained with a multiplex panel of protein antibodies that have a photocleavable indexing oligonucleotide, which enables subsequent readouts. Regions of interest (ROI) were selected on a DSP instrument and illuminated using UV light. Released indexing oligonucleotides from each ROI were collected into designated wells on a microplate for indexing of each ROI for nCounter and readout by direct protein hybridization. ROIs were collected after review by a pathologist to assure the collection of ROIs with invasive cancer cells.

### DSP analysis

NanoString DSP data were normalized using lane-specific External RNA Controls Consortium (ERCC) normalization and sample-specific housekeeping normalization using the controls Ms IgG1, Ms IgG2a, Rb IgG, GAPDH, Histone H3, and S6. Individual observations were filtered to remove outliers and poor-quality samples and were log_2_ transformed for all subsequent analyses. Hierarchical clustering with complete linkage was performed on the 25 proteins with the largest variance across all observations, and principal components were run on the centered and scaled protein expression matrix. Differential protein expression was tested for all noncontrol proteins using linear mixed-effect models with protein expression used as a univariate response, a single categorical variable used as a fixed effect covariate, and a patient-specific random intercept term to account for multiple expression measurements taken per patient. Sample sizes reported are the number of patients, not the total number of measurements. Separate models were run for each subset of patients and fixed effects were analyzed. The models were fitted using the R package lme4 (19), and tests for the significance of fixed effects were calculated using the R package lmerTest (20) using *t* tests with a Satterthwaite approximation for degrees of freedom. *P* values were adjusted for multiple testing using the Benjamini–Hochberg procedure. A false discovery rate (FDR) threshold of 5% was used to report differentially expressed proteins, and log_2_-fold changes were calculated based on the un-log_2_ transformed normalized data. DSP trajectories of individual proteins between time points for each patient were calculated by taking the average expression of that protein by patients at each fixed time point. Pearson correlations were calculated between average DSP Ki-67 measurements per patient and shifted log_2_ IHC Ki-67 measurements [log_2_(Ki-67% + 1%)], and two-sided tests of the correlation coefficient were performed. Because previous studies showed that the spatial location of the immune cells may affect the activity and expression profiles of specific immune cell types (21), we compared the immune regions that were proximal and in direct contact to the invasive cancer cells, the immune regions distal to the invasive cancer cells (within the tumor borders) and immune regions that were between (intermediate) these two locations. Principal components analysis did not segregate these spatially different immune regions (*N* = 108) and, therefore, we did not distinguish between these regions in subsequent analyses (Supplementary Fig. S1A).

### IHC studies and TILs analysis

Dual IHC staining of Ki-67 and cytokeratin was conducted on 4-μm FFPE sections, using both Bond Polymer Refine Kit and Bond Polymer Refine Red kit in Leica Bond RX system. The slides were deparaffinized, and heat-mediated antigen retrieval was performed with EDTA buffer (pH 9.0). The slides were incubated with the antibody against cytokeratin (CAM5.2, Cell Marque) at 1:5,000 dilution for 30 minutes at room temperature, and antigen–antibody reaction was visualized with DAB chromogen. The slides were sequentially incubated with anti–Ki-67 antibody (Biocare Medical; cat. #CRM 325, RRID:AB_2721189) at 1:100 dilution for 60 minutes at room temperature and visualized using Fast Red chromogen. Omission of the primary antibody was used as a negative control. Whole slide images were acquired from stained slides using a Vectra 3.0 Automated Quantitative Pathology Imaging System (Akoya Biosciences) and analyzed using Halo Image Analysis platform (Indica Labs). Image annotations were performed by a pathologist, the areas containing invasive carcinoma were included in image analysis. We utilized the Halo image software, employing an algorithm using color deconvolution to separate brown and red chromogenic stains for analysis, trained to identify the invasive tumor cells based on cytokeratin masking, and subsequently completed cell segmentation. The threshold for Ki-67 was set based on the staining intensity of visualization of red in nucleus staining and applied to the whole annotated image; tumor cells with the intensity above the setting threshold were defined as Ki-67–positive. sTILs were analyzed by a pathologist and scored based on the International sTIL working group method (22).

### Cell lines and cell culture

MCF7 (RRID:CVCL_0031), T47D (RRID:CVCL_0553), CAMA-1 (RRID:CVCL_1115), ZR75.1 (RRID:CVCL_0588), and HEK293 (RRID:CVCL_0045) cells were purchased from the ATCC and authenticated using standard short tandem repeat analysis in 2019. Doxycycline (DOX)-inducible ER Y537S mutant and WT-ER cells as well as *ESR1* Y537S knock-in mutant cells were previously generated in MCF7 cells (23). MCF7, CAMA-1, and HEK293 were cultured in DMEM supplemented with 10% FBS, 1% l-glutamine, and 1% penicillin and streptomycin (P/S). T47D and ZR75.1 were cultured in RPMI-1640 supplemented with 10% FBS, 1% l-glutamine, and 1% P/S. All cells were used at low passage numbers and were tested for *Mycoplasma* using the MycoAlert Mycoplasma Detection Kit (Lonza). For hormone-depleted (HD) conditions, cells were kept in phenol red–free medium supplemented with 10% heat-inactivated charcoal-stripped (CS)-FBS and 1% P/S for 72 hours. All cell lines were incubated at 37°C in 5% CO_2_. To create MCF7_NY-ESO-1 cells, MCF7 cells were transduced with pHAGE vector containing NY-ESO-1 sequence and ZsGreen protein (NY-ESO-1_Luc_ZsGreen_pHAGE). To select NY-ESO-1–expressing cells, MCF7 cells transduced with NY-ESO-1 vectors were sorted twice based on ZsGreen expression. Plasmid NY-ESO-1_Luc_ZsGreen_pHAGE was a gift from Kai Wucherpfening's lab (24). Compounds used for cell lines treatments: beta-E2 (Sigma-Aldrich E2758), fulvestrant (Sigma-Aldrich I4409), birinapant (Selleckchem, 7015), and ARV-471 (Medchem Express LLC, HY-138642).

### Flow cytometry analysis

Treatment conditions for flow cytometry analysis were conducted as follows, unless specified otherwise: Breast cancer cell lines were grown in the presence of E2 10 nmol/L (E2), in hormone-deprived (HD) conditions or treated with either vehicle, fulvestrant, birinapant, or the combination for a total of 72 hours. For IFNγ stimulation studies, after 48 hours, treatments were refreshed with or without IFNγ (10 ng/mL) for last 24 hours prior to flow cytometry analysis. Before the analysis, tumor cells or T cells were suspended in FACS staining buffer [1% BSA, 1 mmol/L EDTA in phosphate-buffered saline (PBS)] and stained with fluorochrome-conjugated antibodies against combinations of the following surface human antigens: HLA-ABC (BioLegend; cat. #311415, RRID:AB_493134), PD-L1/CD274 (BioLegend; cat. #329707, RRID:AB_940358), and HA-tag (BioLegend; cat. #901509, RRID:AB_2565072). Cell viability was determined using propidium iodide exclusion (Life Technologies, P3566) or DAPI (Thermo Fisher, 62248). Flow-cytometric data were acquired using an LSRFortessa cytometer (BD) and analyzed with FlowJo software version 10 (FlowJo LLC).

### Lentivirus production

The day before transfection, HEK293FT cells were plated in 6-well plates at 40% to 60% confluence. Transfection was performed using 6 μL per well of X-tremeGENE HD (Sigma-Aldrich). For each well, 0.4 μg VSVG, 1 μg psPAX2, and 3 μg of the vector of interest were added to 1 mL of Opti-MEM (Life Technologies) along with the transfection agent. After overnight culture, media were changed with 3 mL of DMEM containing 20% FBS for virus collection. The supernatant was harvested after 48 hours and filtered with 0.45 nm filters. Virus particles were either used right away or frozen in aliquots for future transductions.

### T-cell isolation and transduction

PBMCs were isolated from a healthy donor's blood using a density gradient medium (Lymphoprep, STEMCELL Technologies) and SepMate PBMC Isolation Tubes (STEMCELL Technologies). T cells were isolated from PBMCs using the EasySep Human T-cell Isolation Kit (STEMCELL Technologies). Isolated T cells were seeded in nontreated 24-well plates and activated in the presence of 30 IU/ML of IL2 (STEMCELL Technologies) and biotinylated antibodies against human CD2, CD3, and CD28 (Human T-cell Activation/Expansion Kit, Miltenyi) in RPMI-1640 containing 10% FBS, 1% l-glutamine, and 1% P/S and streptomycin.

Freshly activated T cells were transduced with a recombinant T-cell receptor (TCR) specific for the NY-ESO-1 antigen (NY-ESO-1:157–165 epitope) presented in an HLA-A* 02-restricted manner. Briefly, between 0.5 and 1 million activated T cells were plated in nonculture treated 24-well plates in the presence of 30 IU/mL of IL2 and 8 μg/mL of polybrene. Viral particles were added to the mixture and cells underwent spin infection (800G for 2 hours at 37°C. After spin infection, T cells were incubated with viral particles for 3 days. NYESO TCR expression was measured by flow cytometry after 5 days using HA-tag antibody (BioLegend cat. #901509, RRID:AB_2565072) and sorted for coculture experiments. Activated TCR^+^ T cells were then cultured at a density of 0.7–1.0 × 10^6^ cells per mL for 10 to 14 days and used for coculture experiments. All PBMCs and lymphocytes used were obtained from leukapheresis collars from healthy donors at Crisom Core at BWH with an IRB-approved protocol (DFCI protocol 17-684). Recombinant TCR specific for the NY-ESO-1 antigen was a gift from Kai Wucherpfening's lab.

### Chromatin immunoprecipitation sequencing

Chromatin immunoprecipitation (ChIP) experiments were conducted as described previously (23) and were done in duplicates. MCF7 cells were grown in HD conditions for three days (HD) or in the presence of 10 nmol/L of E2 for three days (E2) with or without IFNγ stimulation (10 ng/mL) for 1 hour. Chromatin from approximately 1×10^7^ fixed cells was sonicated to a size range of 200 to 300 bp in a Covaris E220 instrument in 1 mL AFA Fiber milliTUBEs. Solubilized chromatin was subjected to immunoprecipitation overnight with RELA antibody (Cell Signaling Technology; cat. # 8242, RRID:AB_10859369) and bound to protein A and protein G beads (Life Technologies). A fraction of the sample was not exposed to antibody to be used as control (input). The samples were reversed crosslinked, treated with proteinase K, and DNA was extracted. DNA was then submitted for library preparation and sequencing to the Molecular Biology Core Facilities at Dana-Farber Cancer Institute. ChIP-seq analysis was performed as previously reported (23, 25). ChIP reads were aligned to the hg19 genome assembly using BWA (26) and ChIP-seq peaks were called using MACS 2.0 (27, 28). For differential analysis, bed-files were merged using bedops (29) and then DEseq2 was used to assign differential intensities and statistics. Differential binding sites were determined by filtering out insignificant peaks (adjusted *P* < 0.05) and then determining log_2_ FC values between samples for each region using a log_2_ FC >0 or <0. Unsupervised sample-to-sample correlation analysis was done using Euclidean distance and Ward's method. Analysis was done using the CoBRA workflow (30), which included motif analysis.

### ATAC sequencing

Transposase-accessible chromatin sequencing (ATAC-seq) was performed to study global chromatin accessibility for non-IFNγ–stimulated conditions, MCF7 cells were grown in estrogen-deprived conditions (HD) or in the presence of E2 10 nmol/L for 3 days. For IFNγ-stimulated conditions, MCF7 cells were grown in HD or in the presence of E2 10 nmol/L for 48 hours and then treated with 10 ng/mL of IFNγ for 24 hours. ATAC-seq was performed as previously described (31, 32). Briefly, cells were resuspended in 1 mL of cold ATAC-seq resuspension buffer (RSB; 10 mmol/L Tris-HCl pH 7.4, 10 mmol/L NaCl, and 3 mmol/L MgCl2 in water) and centrifuged. Cell pellets were then resuspended in ATAC-seq RSB (0.1% NP40, 0.1% Tween-20, and 0.01% digitonin) and incubated on ice. After lysis, ATAC-seq RSB containing 0.1% Tween-20 (without NP40 or digitonin) was added. Nuclei were centrifuged and then were resuspended in 50 μL of transposition mix (25 μL 2× TD buffer, 2.5 μL transposase (100 nmol/L final), 16.5 μL PBS, 0.5 μL 1% digitonin, 0.5 μL 10% Tween-20, and 5 μL water). Transposition reactions were incubated at 37°C for 30 minutes. Reactions were cleaned up with Zymo DNA Clean and Concentrator 5 columns. For ATAC-seq data analysis, we used Burrows–Wheeler Aligner (BWA; ref. 33) to map sequencing reads to the reference genome and MACS2 (27) for peak calling. DESeq2 (34) was applied to identify the differentially accessible regions with or without IFNγ treatment from ATAC-seq data, and significant differences were based on a log_2_ FC >0.5 or <0.5, *q* < 0.05.

### RNA sequencing

MCF7 cells were grown in hormone-deprived conditions or in the presence of E2, 10 nmol/L for 72 hours. RNA was extracted after 6, 12, 24, and 48 hours of IFNγ stimulation. For each experiment, total RNA was isolated using an RNeasy Mini Kit (Qiagen, 74134) in triplicates. RNA concentrations were measured by NanoDrop and the quality of RNA was determined by a Bioanalyzer. For all cell line studies, samples were analyzed in at least duplicates. RNA-seq libraries were made using the TruSeq RNA Sample Preparation Kit (Illumina). Samples were sequenced on an Illumina Nextseq500. The RNA-seq analyses were performed using the VIPER analysis pipeline (35, 36). Alignment to the hg19 human genome was done using STAR v2.7.0f followed by transcript assembly using cufflinks v2.2.1 (37) and RseQC v2.6.2 (38). Differential expression analysis was done using DEseq2 v1.18.1 (34). Significant changes were based on a log_2_ FC > 0.5 or <0.5, adj-*P* < 0.05, DEseq2 gene set enrichment analysis (GSEA) was performed using the Broad GSEA Application (39). For The Cancer Genome Atlas (TCGA) analyses, we used gene set variation analysis, which is a nonparametric method for estimating the variation of pathways over a sample population in an unsupervised manner (40).

### Binding and expression target analysis

To identify the putative genes that are immediate transcription targets of RELA, we used the binding and expression target analysis (BETA) tool (41). This tool consists of three tools, including BETA minus, BETA basic, and BETA plus. In this article, we used BETA minus and BETA basic. In BETA minus, transcription factor target gene prediction is based on a regulatory score applying ChIP-seq data only. In BETA basic, transcription factor activating or repressive function is predicted based on ChIP-seq and RNA-seq data using the Kolmogorov–Smirnov test. For no IFNγ conditions, we used RNA-seq and RELA ChIP-seq data from MCF7 cells grown in HD or E2 for 3 days. For IFNγ conditions, we used RNA-seq data from MCF7 cells grown in HD or E2 for 3 days stimulated with IFNγ for 6 hours and RELA ChIP-seq data from MCF7 cells grown in HD or E2 for 3 days stimulated with IFNγ for 1 hour. The RelA_HD gene set was derived by overlapping the genes that were identified by BETA basic to be regulated by RelA in HD conditions and are upregulated in HD conditions compared with E2 conditions by RNA-seq (log_2_ FC > 0.5, adj-*P* <0.05, DEseq2).

### RELA CRISPR knockouts

To validate the role of RELA in the response to IFNy, we performed CRISPR-Cas9–mediated deletion of RELA in multiple cell lines. Briefly, Cas9-expressing cell lines (RRID:Addgene_68343) were transduced with a guide RNA (gRNA) designed to match RELA exon sequence (RRID:Addgene_67974) or with the empty vector as a control. We created two different knockout (KO) cellular populations using two different gRNAs for RELA (RELA KO1 and RELA KO2). Knockouts were validated by mRNA and protein levels.

RELA KO1 guideRNA: GAAGATCTCATCCCCACCG

RELA KO2 guideRNA: CTACGACCTGAATGCTGTG

### Cytokine profiling

Multiplex assays were performed utilizing the bead-based immunoassay Human Cytokine/Chemokine Magnetic Bead Panel (catalog no. HCYTMAG-60K-PX30) on a Luminex MAGPIX system (Merck Millipore). Conditioned media concentration levels (pg/mL) of each protein were derived from five-parameter curve-fitting models. Lower and upper limits of quantitation were imputed from standard curves for cytokines above or below detection. For non-IFNγ–stimulated conditions: MCF7 cells and MCF7 *ESR1* Y537S knock-in mutant cells were grown in estrogen-deprived conditions (HD) or in the presence of E2 10 nmol/L (E2) for 3 days. For IFNγ-stimulated conditions, MCF7 cells and MCF7 *ESR1* Y537S knock-in mutant cells were grown in estrogen-deprived conditions (HD) or in the presence of 10 nmol/L E2 for 72 hours and stimulated with 10 ng/mL of IFNγ for the last 24 hours.

Proteome Profiler Human XL Cytokine Array Kit (ARY022B; R&D Systems) was used to analyze levels of cytokines, chemokines, and growth factors from conditioned media of breast cancer cells following the manufacturer's instructions. MCF7 cells were pretreated with vehicle, fulvestrant (10 nmol/L), birinapant (100 nmol/L) and the combination of both drugs for 48 hours, FBS-containing media were then removed, and IFNγ was added along with the treatments in an FBS-free media for 24 hours along when conditioned media were collected.

### Patient-derived xenograft studies

Female 5–6-week-old NRG mice (Jackson labs stock #007799) were implanted with HCI-011 patient-derived xenograft (PDX) tumor fragments using a previously described protocol with estrogen supplementation (42). When tumors reached approximately 100 mm^3^, mice were randomized into treatment or control groups. Mice received either vehicle control (15% captisol IP, 3×/week for 5 weeks), birinapant (20 mg/kg IP, 3×/week for 5 weeks), fulvestrant (200 mg/kg subQ, 1×/week for 5 weeks), or the combination of birinapant and fulvestrant at the same doses as single agents. For birinapant treatment, birinapant (Medchem Express, HY-16591) was dissolved in 15% captisol at a concentration of 2 mg/mL (made fresh every time), and 100 μL/10 g mouse body weight was injected intraperitoneally. For fulvestrant treatment, 250 mg of fulvestrant (Selleck Chemicals, S1191) was dissolved in 0.5 mL DMSO, incubated at 37°C for 15 minutes and then diluted 1:20 with corn oil to give a final concentration of fulvestrant of 25 mg/mL (made fresh every time). 80 μL/10 g mouse body weight was injected subcutaneously. Animal experiments were all conducted in compliance with institutional guidelines and regulations after approval from the University of Utah Institutional Animal Care and Use Committee, under protocol 21-06008.

### T-cell coculture assay

MCF7 NY-ESO-1 and primary T cells expressing NY-ESO-1 TCR were used for cocultured assays. One day before coculture, MCF7 NY-ESO-1 cells were seeded at a specific density on 24-well plates in the same media as the T cells and treated overnight with the specified conditions. The next day, NY-ESO-1_TCR^+^ T cells were added to the plates at a 1:1 effector:target ratio and cocultured for 12–16 hours. Thereafter, T cells were carefully washed away by 2× PBS washes. Hoechst was used for nuclear staining and propidium iodide was used for staining dead cells. Tumor cells were counted using the Celigo image cytometer instrument (Nexcelom, RRID:SCR_018808).

### Proliferation assays

Breast cancer cells were plated in 24-well plates at a density of 8,000 cells per well. At indicated time points, cells were counted using the Celigo image cytometer (Nexcelom, RRID:SCR_018808). Hoechst was used for nuclear staining and propidium iodide was used for staining dead cells. Fulvestrant and birinapant were used for the growth and synergy studies. Synergy score was calculated using SynergyFinder and ZIP synergy scoring (43).

### Immunoblotting

Cells were lysed in RIPA buffer (Thermo Fisher, 89900) supplemented with phosphatase and protease inhibitors and subjected to SDS-PAGE. Antibodies used were ERa (Santa Cruz Biotechnology; cat. #sc-543, RRID:AB_631471), RelA/p65 (Cell Signaling Technology; cat. #6956, RRID:AB_10828935), phospho-RelA Ser536 (Cell Signaling Technology; cat. #3033, RRID:AB_331284), GAPDH (Santa Cruz Biotechnology; cat. #sc-25778, RRID:AB_10167668), RelB (Cell Signaling Technology; cat. #10544, RRID:AB_2797727), NF-κB1 p105/50 (Cell Signaling Technology; cat. #12540, RRID:AB_2687614), NF-κB2 p100/52 (Cell Signaling Technology cat. #3017, RRID:AB_10697356), IRF1 (Cell Signaling Technology; cat. #8478, RRID:AB_10949108), STAT1 (Cell Signaling Technology; cat. #9176, RRID:AB_2240087), phospho-STAT1 Tyr701 (Cell Signaling Technology; cat. #9167, RRID:AB_561284), MHC-I (Thermo Fisher Scientific; cat. #MA5-11723, RRID:AB_10985125), PD-L1 (Abcam; cat. #ab228415, RRID:AB_2884993).

### Three-dimensional migration assay

Immune cell infiltration was assessed as previously described (44). Briefly, MCF7 cell spheroids were generated by seeding 5 × 10^5^ cells in suspension in an Ultra-low attachment dish for 48 hours. MCF7 spheroids were treated for 48 hours with vehicle (DMSO), fulvestrant (10 nmol/L), birinapant (100 nmol/L), and the combination. Samples were then pelleted and resuspended in type I rat tail collagen (Corning) at a concentration of 3 mg/mL following the addition of 10× PBS with phenol red and pH adjustment using NaOH. pH 7.0–7.5 was confirmed using PANPEHA Whatman paper (Sigma-Aldrich). Cells and collagen were kept on ice to prevent polymerization. The spheroid-collagen suspension was then injected into the central gel region of the three-dimensonal (3D) DAX-1 3D microfluidic cell culture chip (AIM Biotech). Microfluidic devices were utilized as previously described, with a central region containing the cell–collagen mixture in a 3D microenvironment (30,000 cells in 10 μL), flanked by two media channels located on either side. After injection, collagen hydrogels containing cells were incubated for 40 minutes at 37°C in humidity chambers, then hydrated with culture media, with labeled CD8^+^ T cells (E:T ratio 2:1) added to one of the side channels. CD8^+^ T cells were labeled with CellTrace Red Stain (Thermo Fisher Scientific) following the manufacturer's instructions. Treatments were refreshed along with the culture media. After 72 hours of incubation, images were captured on a Nikon Eclipse 80i fluorescence microscope equipped with Z-stack (Prior) and CoolSNAP CCD camera (Roper Scientific). Image capture and analysis were performed using the NIS-Elements AR software package. Whole device images were achieved by stitching in multiple captures. Quantification of immune cell infiltration into the 3D tumor microenvironment was performed by measuring the total cell area of cell tracker dye in the entire gel region.

### Data availability

The whole-genome sequencing, RNA-seq, ChIP-seq, and ATAC-seq data were all submitted to GEO accession number GSE214054. RNA-seq and clinical data from the TCGA breast cancer cohort (45) were downloaded from https://portal.gdc.cancer.gov/ and reference (46) for the breast cancer biopsies pre- and postneoadjuvant aromatase inhibitor (AI) treatment. All other raw data are available upon request from the corresponding author.

## Results

### ET in HR^+^ primary breast cancer increases the expression of B2M and STING

**Figure 1.**
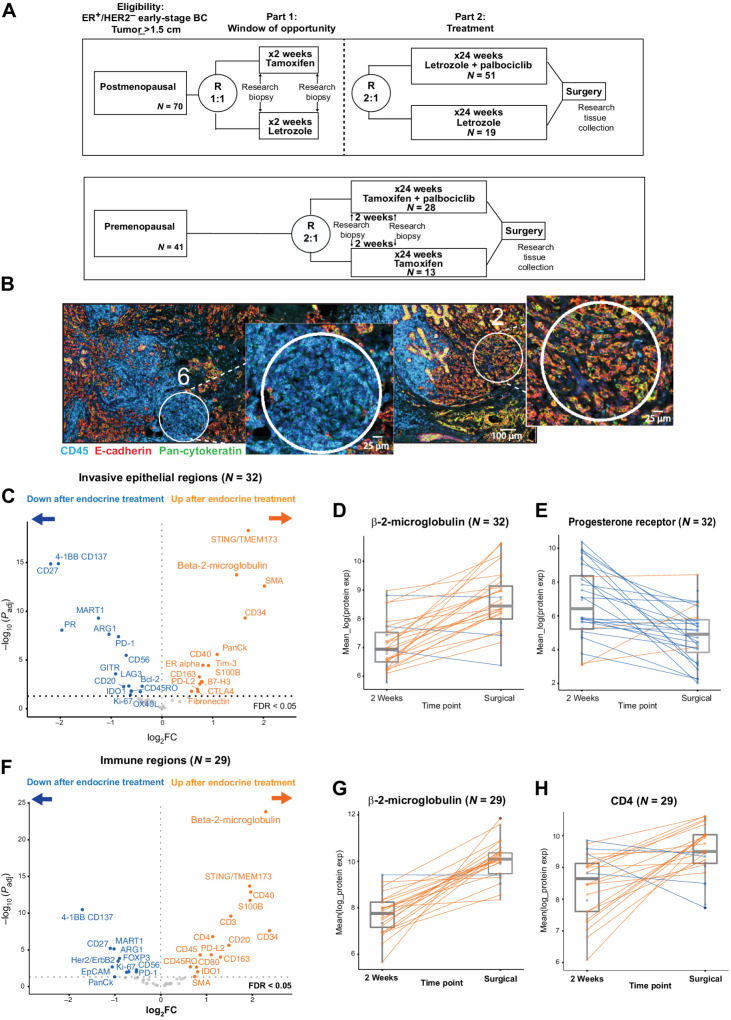
Digital spatial analysis. **A,** Schema of the endocrine therapy for PELOPS (NCT02764541). Numbers (*N*) represent the number of tissue samples included in the digital spatial profiling. **B,** Representative images of immunofluorescence staining for the regions of interest. CD45, cyan; E-cadherin, red; pan-cytokeratin, green. The magnified panels represent two ROIs; immune (#6) and invasive epithelial cells (#2). **C–E,** Volcano plot of log_2_-fold changes and adjusted *P* values of tests for differential protein expression comparing pre and post 24 weeks of endocrine treatment (2 weeks vs. surgical) within invasive epithelial cellular regions (**C**) and corresponding individual average patient trajectories for β2-microglobulin (**D**) and PR (**E**). **F–H,** Volcano plot of log_2_-fold changes and adjusted *P* values of tests for differential protein expression comparing pre and post 24 weeks of endocrine treatment (2 weeks versus surgical) within immune regions (**F**) and corresponding individual mean patient trajectories for β2-microglobulin (**G**) and CD4 (**H**). Horizontal dotted line denotes a 5% FDR threshold.

To investigate the effects of ET on the TME and the invasive cancer epithelial cells (ICEC) in HR^+^ breast cancer, we performed multiplex spatial proteomic characterization of primary breast cancers. We assayed 294 primary breast cancer tissue samples from 111 patients with primary ER^+^/HER2-negative breast cancer who participated in the neoadjuvant PELOPS, including samples from baseline, on treatment (day 15), and surgery time points (Fig. 1A). The clinicopathologic characteristics of the patients are detailed in Supplementary Table S1. ROIs were selected based on geographical and phenotypic characteristics. Pan-cytokeratin and CD45 immunofluorescence was used to select regions of ICECs and immune regions (regions of sTIL) for the quantification of tumor and immune-related proteins (Fig. 1B; Supplementary Table S2 and S3). Unsupervised hierarchical clustering of all ROIs to look for broad patterns within and between patients segregated the immune from ICECs regions (Supplementary Fig. S1B). Comparison of the immune and ICECs ROIs at the 3 time points showed upregulation of cell-surface markers of T and B lymphocytes in the immune regions, whereas ICECs regions exhibited upregulation of ER, Pan-CK, EpCAM, and progesterone receptor (PR; Supplementary Fig. S1C–S1F; Supplementary Table S4).

Next, we examined the effects of ET. In the postmenopausal patients randomized to the window of opportunity part of the trial, after two weeks of tamoxifen or letrozole treatment there were no significant expression changes in the sTIL regions. In contrast, in the ICECS regions, tamoxifen treatment resulted in a significant increase in PR and a trend toward a decrease in Ki-67 (Supplementary Fig. S1G–S1H; Supplementary Table S4), whereas letrozole treatment led to a significant decrease in Ki-67 and PR (Supplementary Fig. S1I–S1J; Supplementary Table S4). This is consistent with previous studies showing greater suppression of Ki-67 with an AI compared with tamoxifen after two weeks of treatment (47). Of note, there was a strong correlation between Ki67 levels and the ratio of Ki-67 at 2 weeks/baseline comparing Ki-67 quantified by DSP and IHC (Supplementary Fig. S1K–S1N). Thus, these results provide evidence that the analysis of selected regions can represent the entire section.

After 24 weeks of ET, we detected significant changes in the expression of several immune-related proteins in the ICEC regions, including upregulation of STING (stimulator of interferon genes), B2M, CD40, TIM3, B7-H3, and CTLA4, and downregulation of TNFR and CD137 (Fig. 1C–E; Supplementary Table S5). In addition, we observed an increase in ER expression, likely an adaptative response to ER signaling blockade, and a decrease in PR and Ki-67 (Fig. 1E). In the immune regions (Fig. 1F–H; Supplementary Table S6), there was also a significant increase in B2M, STING, and CD40 and decrease in CD137. Additionally, there were multiple changes unique to the immune regions, such as an increase in the CD3 and the CD4 T-cell markers and a decrease in the regulatory T-cell (Treg) marker FOXP3 (Supplementary Fig. S2A–S2F).

To validate our results in an independent patient cohort, we turned to the transcriptomic data from the NEOAI study, a retrospective study of patients with primary ER^+^/HER2^−^ breast cancer who received neoadjuvant treatment with an AI for at least 4 weeks (48, 49). Thirty-two of the proteins tested in our study overlapped with the NanoString mRNA panel that was applied in NEOAI (Supplementary Table S7). Because the majority (63%) of these proteins were significantly upregulated in the immune regions compared with the cancer cell regions, we used the expression levels of the immune regions. Indeed, we observed a significant correlation between the gene-expression changes after AI treatment in the NEOAI study and the protein expression changes after letrozole treatment in PELOPS (Supplementary Fig. S2G).

**Figure 2.**
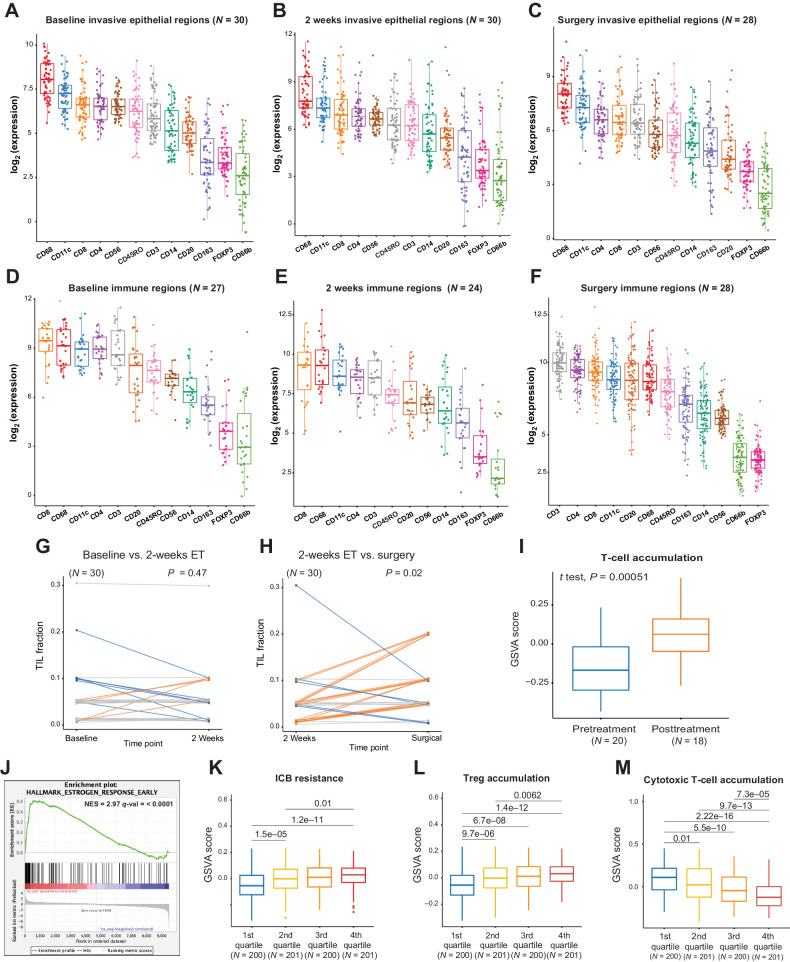
Endocrine treatment shapes tumor immune microenvironment in primary hormone receptor–positive breast cancer. **A–F,** Digital spatial profiling proteomic levels (log_2_ expression levels) of immune cell surface markers ranking from highest to lowest expression. **A,** Baseline levels within the immune regions in tumors from patients who received endocrine therapy [F(11, 312) = 57.1, *P* < 2e−16, one-way ANOVA]. **B,** Protein expression levels within the immune regions after 2 weeks of endocrine treatment [F(11, 276) = 41.7, *P* < 2e−16, one-way ANOVA]. **C,** Protein expression levels within the immune regions after 24 weeks of endocrine treatment at the time of surgery [F(11, 1212) = 305.2, *P* < 2e−16, one-way ANOVA]. **D,** Baseline levels within the invasive epithelial cell regions in tumors from patients who received endocrine therapy [F (11, 732) = 126.3, *P* < 2e−16, one-way ANOVA]. **E,** Protein expression levels within the invasive epithelial regions after 2 weeks of endocrine treatment (*P* < 2e−16, one-way ANOVA). **F,** Protein expression levels within the invasive epithelial cell regions after 24 weeks of endocrine treatment at the time of surgery (*P* < 2e−16, one-way ANOVA). Boxplots show median, 25th, and 75th percentiles as boxes, the minimum of the 75th percentile + 1.5 × IQR, and the maximum observation as the upper whisker and the maximum of the 25th percentile −1.5 × IQR and the minimum observation as the lower whisker. **G,** Trajectory plot of TIL fractions between baseline and 2 weeks. *P*-val, *P* value (paired Wilcoxon signed-rank test with continuity correction). **H,** Trajectory plot of TIL fractions between 2 weeks and surgery for all patients given endocrine treatment that have TIL observations at all three time points. Each trajectory corresponds to a single patient. *P*-val, *P* value (paired Wilcoxon signed-rank test with continuity correction). **I,** GSVA of the T-cell accumulation gene set in primary ER^+^ breast cancer biopsies from pre- and post-neoadjuvant AI treatment. **J,** Enrichment plot of the top-ranked gene set (estrogen response) enriched in the ESR1 highest (fourth quartile) versus ESR1 lowest (first quartile) ER–positive breast cancer samples from the TCGA cohort. **K–M,** GSVA of RNA-seq from the ER–positive breast cancer samples from TCGA divided into quartiles based on ESR1 mRNA levels testing the enrichment score (*y*-axis) for signatures of immune-checkpoint blockade (ICB) resistance (**K**), T-regulatory (Treg) accumulation (**L**), and cytotoxic T-cell accumulation (**M**). Comparison between the quartiles was done with a *t* test. *N*, number of patients included in the corresponding analysis.

To assess the relative abundancies of the immune cell populations and how they are affected by ET, we focused on the expression levels of immune cell-surface markers. Analysis of the ICECs and the immune regions separately at each of the three time points revealed unequal expression of the immune cell surface markers within each region subtype (Fig. 2A–F) and differences between the ICECs and the immune regions. In the ICECs regions at baseline, the most abundant markers of immune cells infiltrating the tumor cells were the pan-macrophage (CD68) and dendritic cell (CD11c) markers, whereas the least abundant were markers of Tregs (FOXP3) and active neutrophils (CD66b; Fig. 2A). This ranking persisted after 2 and 24 weeks of ET (Fig. 2B and C). In the immune regions at baseline (Fig. 2D) and after 2 weeks of ET (Fig. 2E), the most abundant immune cell-surface marker was the CD8 T-cell marker, followed by the CD68 macrophage marker, which is consistent with previous studies showing that the most abundant immune cells in breast cancer, including ER^+^ breast cancer, are T cells and myeloid cells (50). After 24 weeks of ET, the most abundant immune cell-surface markers were CD3 and CD4, followed by CD8 (Fig. 2F). CD4^+^ T cells are important for the function of CD8^+^ T cells (51), and a higher CD4:CD8 ratio has been shown to be important in sustaining the function of adoptively transferred T cells (52), suggesting that this change may favorably affect immunogenicity.

As expected in ER^+^ breast cancers, the median fraction of sTIL in the entire slide was low (Supplementary Table S1). Although there were no changes in the fraction of sTIL after two weeks of ET (Fig. 2G), after 24 weeks of treatment at the time of surgery there was a significant increase in the fraction of sTIL (Fig. 2H). However, the sTIL fraction was still relatively low at this time point (<0.3). The increase in sTIL and the changes in the expression of immune-related proteins did not correlate with Ki67 suppression at two weeks, a biomarker of response to ET, suggesting that these changes were not dependent on tumor response to treatment (Supplementary Fig. S2H–S2I). In keeping with the increase in the sTIL fraction that we detected, in a separate cohort of ER^+^/HER2-negative primary BCs for which RNA-seq data were available (46), neoadjuvant treatment with an AI resulted in an increase in the expression of the gene signature of cytotoxic T-cell accumulation (Fig. 2I; ref. 53).

To study the associations between ER signaling and immune-related pathways in a separate cohort of ER^+^ breast cancers, we utilized the bulk transcriptomic data from TCGA. We divided the ER^+^ breast cancers (*n* = 802) to quartiles based on *ESR1* mRNA levels. GSEA comparing tumors in the first versus fourth quartile confirmed that the tumors with higher expression of *ESR1* mRNA levels exhibited higher ER transcriptional activity (Fig. 2J). We next tested the association between *ESR1* mRNA levels and the expression of signatures that predict benefit from ICIs applying gene set variation analysis (GSVA; ref. 40). High *ESR1* mRNA was associated with increased expression of a signature of ICI resistance (Fig. 2K; ref. 54) and Treg accumulation (Fig. 2L; ref. 54). Conversely, lower *ESR1* mRNA expression was associated with increased cytotoxic T-cell accumulation (Fig. 2M; ref. 53). These findings are consistent with the analysis of the PELOPS biopsies, where we observed increased expression of B2M and CD4, increased sTIL fraction, and decreased expression of FOXP3 after ET.

Previous studies demonstrated that CDK4/6i can increase the immunogenicity and response to ICIs in models of breast cancer and other cancer types by influencing the tumor cells and TME through several mechanisms (55, 56). In our study, we also analyzed the effects of 24 weeks of treatment with palbociclib and ET in HR^+^ breast cancers. The most significant changes at the protein level in the ICECS regions (*N* = 79) included upregulation of STING, B2M, and the immune checkpoint TIM3 (Supplementary Fig. S3A). In the immune regions (*N* = 77), the most significant changes were increased expression of B2M and CD40 (Supplementary Fig. S3B). Comparison of the protein changes induced by ET alone versus ET in combination with palbociclib revealed a high overlap, and nearly all proteins upregulated by ET were also upregulated by the treatment combination. In contrast, there were additional proteins uniquely upregulated by the addition of palbociclib, including granzyme B, PD-L1, HLA-DR, and others (Supplementary Fig. S3C–S3D). Several proteins were uniquely downregulated in ET alone, such as FOXP3, PD-1, and IDO-1 (Supplementary Fig. S3E–S3F). Taken together, these results indicate that although the effects of ET as monotherapy and in combination with palbociclib differ, both treatments affect the innate and adaptive immune pathways in a manner that overall favors increased immunogenicity.

### Estrogen receptor signaling impedes MHC-I expression

**Figure 3.**
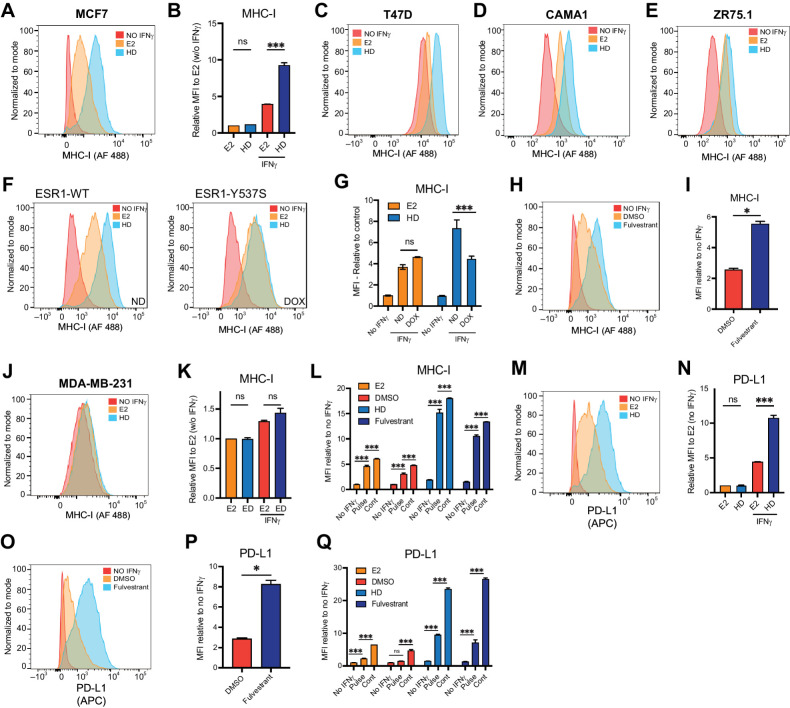
E2 modulates response to IFNγ stimulation. **A,** Histogram of MHC-I levels assessed by flow cytometry following 3 days of E2 stimulation or HD in the presence or absence of IFNγ (10 ng/mL) for the last 24 hours in MCF7 cells. **B,** Quantification of median fluorescence intensity (MFI) of MHC-I from **A**. Values are normalized to E2 stimulation with vehicle control (no IFNγ) cells. **C–E,** MHC-I levels assessed by flow cytometry following 3 days of E2 stimulation or HD in the presence or absence of IFNγ (10 ug/mL) for the last 24 hours in ER^+^ T47D cells (**C**), CAMA1 cells (**D**), and ZR75.1 (**E**). **F** and **G,** Histograms (**F**) and MFI quantification (**G**) of MHC-I levels following 3 days of E2 stimulation or HD in the presence or absence of IFNγ (10 ng/mL) for the last 24 hours in MCF7 cells expressing the ESR1 Y537S mutation induced by DOX treatment cells. Two-way ANOVA. **H,** Histogram of MHC-I levels assessed by flow cytometry following 3 days of vehicle or fulvestrant (10 nmol/L) treatment in the presence or absence of IFNγ (10 ng/mL) for the last 24 hours in MCF7 cells. **I,** MFI quantification of MHC-I from **H**. Values are normalized to vehicle control (no IFNγ) cells. Error bars, mean ± SD of at least two replicates. *, *P* < 0.05, paired *t* test. **J,** Histogram of MHC-I levels assessed by flow cytometry following 3 days of E2 stimulation or HD in the presence or absence of IFNγ (10 ng/mL) for the last 24 hours in ER-negative MDA-MB-231 cells. **K,** MFI quantification of MHC-I from **J** values is normalized to E2 stimulation (no IFNγ) cells. **L,** MFI quantification of MHC-I levels assessed by flow cytometry of cells grown in HD, E2 conditions, or treated with fulvestrant or DMSO for 72 hours in the absence of IFNγ (no IFNγ) or with a 15-minute treatment of IFNγ (10 ng/mL) 24 hours prior flow analysis (Pulse) or for the last 24 hours (continuous, cont) prior to flow cytometry analysis. **M,** PD-L1 levels assessed by flow cytometry following 3 days of E2 stimulation or HD in the presence or absence of IFNγ (10 ng/mL) ×24 hours in MCF7 cells. **N,** MFI quantification of MHC-I from **M**. **O,** PD-L1 levels assessed by flow cytometry following 3 days of vehicle or fulvestrant (10 nmol/L) treatment in the presence or absence of IFNγ (10 ng/mL) ×24 hours in MCF7 cells. **P,** MFI quantification of MHC-I from **O**. *, *P* < 0.05. **Q,** MFI quantification of PD-L1 levels assessed by flow cytometry after treatment for 72 hours in the absence of IFNγ (no IFNγ), with a 15-minute treatment of IFNγ (10 ng/mL) 24 hours prior to flow cytometry analysis (Pulse) or for the last 24 hours (cont) prior to flow cytometry analysis. Statistics for the panel, if not mentioned differently, are ***, *P* < 0.001; n.s., not significant. Error bars, mean ± SD of at least two replicates. Two-way ANOVA.

Because the upregulation of B2M in the ICEC regions in ER^+^ breast cancers was among the most significant changes after ET, we sought to investigate the cancer cell–intrinsic effects of ER perturbation on MHC-I expression in ER^+^ breast cancer cell lines (MCF7, T47D, CAMA1, and ZR75.1). To this end, ER^+^ breast cancer cells were cultured in hormone-deprived (HD) conditions or in the presence of E2 (10 nmol/L) for 72 hours, with IFNγ stimulation during the last 24 hours prior to flow cytometry analysis of MHC-I levels (Supplementary Fig. S4A). Baseline levels of MHC-I varied between the different cell lines, but in all cells, the expression was induced by IFNγ stimulation. Strikingly, the IFNγ induced upregulation of MHC-I was lower in E2-treated compared with HD conditions in all cell lines (Fig. 3A–E). These results were confirmed by Western blot analysis, which demonstrated that MHC-I expression was detectable in a dose-dependent manner after 24 hours of IFNγ stimulation only in HD conditions (Supplementary Fig. S4B). To determine that these results were through the inhibition of ER, we tested MCF7 cells with DOX-induced expression of the *ESR1* Y537S-activating mutation that engenders ligand-independent ER activity. In response to IFNγ stimulation, MHC-I expression in HD conditions was significantly lower in cells that expressed the Y537S mutation compared with the isogenic cells without the expression of the Y537S mutation and was comparable with the level of MHC-I detected in E2-treated conditions in the *ESR1*-WT and *ESR1*-Y537S mutant cells (Fig. 3F–G). In contrast, treatment with the selective estrogen degrader, fulvestrant, or the novel ER degrader, ARV-471 (57), which have activity in the presence of the ER mutations, resulted in increased MHC-I expression (Supplementary Fig. S4C and S4D). Furthermore, treatment with fulvestrant also resulted in enhanced MHC-I expression after IFNγ stimulation in the presence of WT-ER (Fig. 3H and I). Lastly, hormone deprivation did not influence IFNγ-induced MHC-I expression in the MDA-MB-231 breast cancer cells that do not express ER (Fig. 3J and K). Because previous *in vivo* studies showed that T cells produce a rapid single pulse of IFNγ (58), we tested the MHC-I expression in response to a 15-minute pulse of IFNγ. HD and fulvestrant treatment led to a robust increase in MHC-I expression in response to a pulse of IFNγ that was comparable with continuous treatment with IFNγ in HD conditions and fulvestrant treatment (the ratio of MHC-I levels between pulse and continuous IFNγ after HD and fulvestrant was 1.18 and 1.27, respectively; Fig. 3L). Because we also observed a strong increase in the expression of STING in primary ER^+^ breast tumors in the regions of immune and invasive epithelial cancer cells in response to ET, we tested STING levels in the MCF7 cells. In contrast to our findings in the tumor samples (Fig. 2I), we did not detect STING expression at baseline or after IFNγ stimulation in HD or E2 conditions in MCF7 cells (Supplementary Fig. S4E). Thus, as opposed to the increase in MHC-I expression in response to ET and IFNγ stimulation that is, at least in part, a cell-intrinsic effect and observed in the breast cancer cells in 2D culture, the effect of ET on STING expression is likely not cell intrinsic, but rather dependent on a more complex interplay between the cancer cells and the TME.

Although PD-L1 expression is immunosuppressive, it is a key target of ICIs and its expression is stimulated by IFNγ. We, therefore, tested the impact of ET on IFNγ-induced PD-L1 levels. Like MHC-I expression, HD and fulvestrant treatment increased PD-L1 expression in response to IFNγ (Fig. 3M–P). Moreover, the effect of HD on the expression of PD-L1 was not seen in *ESR1*-Y537S mutant or ER-negative MDA-MB-231 cells (Supplementary Fig. S4F–S4J). However, in contrast to the regulation of MHC-I, the increase in PD-L1 expression in response to HD and fulvestrant treatment was greater with continuous IFNγ stimulation compared with the short pulse of IFNγ (the ratio of PD-L1 levels between continuous and pulse IFNγ stimulation in HD and fulvestrant treatment was 2.46 and 3.73, respectively; Fig. 3Q), suggesting that ET may not have a significant impact on PD-L1 expression in physiologic conditions. Likewise, in the PELOPS trial, we did not detect a significant change in PD-L1 levels after 2 or 24 weeks of ET.

### ER inhibition enhances the response to IFNγ stimulation

**Figure 4.**
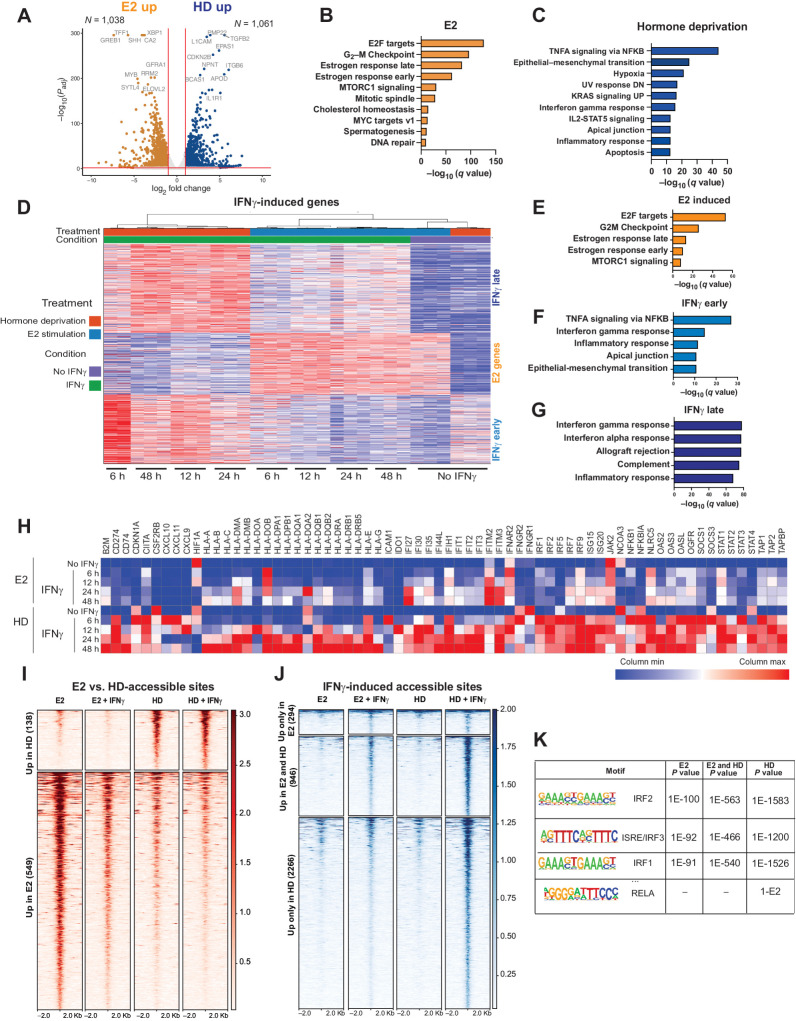
Estrogen deprivation upregulates IFNγ response through NF-κB signaling. **A,** RNA-seq analysis of MCF-7 cells grown in HD conditions or in the presence of E2 for three days. The volcano plot shows genes differently expressed between HD- and E2-treated conditions (log_2_FC>1, *P*_adj_ < 0.01). Number on the top shows the total number of genes differentially expressed for each condition. **B,** GSEA of upregulated pathways in E2-stimulated cells. **C,** GSEA of upregulated pathways in the HD condition. **D,** Three-cluster K-means plot of genes without and with IFNγ stimulation at different time points in cells grown in HD or E2 conditions with and without E2 for three days. **E–G,** Hallmark pathway analysis of “E2-induced” genes (**E**), “IFNγ early” (**F**), and “IFNγ late” (FDR < 0.05; **G**). **H,** Supervised heat map of the expression of IFNγ and antigen presentation-related genes in E2-treated and HD condition without and with IFNγ treatment. **I–J,** ATAC-seq that was performed in MCF7 cells grown in the presence or absence of E2 for 48 hours and followed by ± IFNγ 10 ng/mL stimulation for 24 hours. **I,** Tornado plots of chromatin accessibility sites based on ATAC-seq showing accessible sites significantly different between E2 and HD conditions showing IFNγ-treated or no IFNγ-treated conditions. **J,** Tornado plots showing the chromatin sites accessibly induced by IFNγ stimulation in E2 versus HD conditions. **K,** Motifs enriched in the chromatin accessible sites in **J**.

To gain mechanistic insights into how ER inhibition upregulates MHC-I levels, we performed RNA-seq in MCF7 cells in HD and E2 conditions with and without IFNγ stimulation (Supplementary Tables S8 and S9). We first looked at the genes that were differentially expressed in HD versus E2 growth conditions without IFNγ stimulation (Fig. 4A). Hallmark pathway analysis showed enrichment of genes involved in the cell cycle, estrogen response, and mTOR1 signaling in E2 conditions (Fig. 4B). Interestingly, in HD conditions the upregulated genes were enriched in genes related to an immune response. Notably, NF-κB signaling was the most significantly enriched pathway (Fig. 4C). Unsupervised K-means clustering of the transcriptomes when evaluating the effects of IFNγ in a time-dependent manner revealed three main gene groups that we designated as follows: E2-regulated, IFNγ-early, and IFNγ-late genes (Fig. 4D and Supplementary Table S10). The E2-regulated group consists of genes that were upregulated in E2 conditions prior to IFNγ stimulation and remained overexpressed in E2 versus HD conditions after IFNγ stimulation. Pathway analysis showed that these are E2-regulated genes (Fig. 4E). IFNγ-early genes were overexpressed in HD conditions prior to IFNγ stimulation and were further upregulated after IFNγ stimulation with highest expression in HD condition after 6 hours of IFNγ treatment. These genes were enriched in NF-κB signaling and IFNγ signaling (Fig. 4F). The IFNγ-late genes exhibited IFNγ responsiveness in E2 and HD conditions with a higher degree of upregulation in HD conditions. However, prior to IFNγ treatment, these genes had similar expression levels in HD and E2 conditions (Fig. 4G). Overall, there were more genes significantly upregulated and downregulated with IFNγ in HD compared with E2 conditions at all time points (log FC > 1 adj *P* < 0.01; Supplementary Fig. S5A; Supplementary Tables S7 and S8). In keeping with the increase in the protein levels of MHC-I after hormone deprivation and the results of the DSP in the PELOPS cohort, higher transcript levels of B2M were seen in HD conditions. The increase in B2M was detected prior to IFNγ stimulation (HD vs. E2 condition) and was enhanced after 6 and 12 hours of IFNγ stimulation. Additionally, other genes of the MHC-I complex (HLA-A/HLA-B/HLA-C) and of the antigen-presenting machinery (NLRC5, TAP1, and TAP2) were upregulated in HD conditions only after IFNγ stimulation (Fig. 4H). Multiple transcripts directly related to the IFN signaling were also upregulated. Among these genes were the IFNγ receptor heterodimers (IFNGR1 and IFNGR2) that had the highest expression in HD conditions prior to IFNγ stimulation, suggesting that in HD conditions, the cells are already poised to enhance response to IFNγ. In contrast, the expression of interferon regulatory factors (IRF) genes that are downstream of the IFNγ receptors was increased in HD conditions only after IFNγ stimulation (Fig. 4H). Additionally, a gene set (CXCL9, CXCL10, IDO-1, HLA-DRA, and STAT1) that was shown to be predictive of response to ICI had increased expression after IFNγ stimulation in HD conditions only (59).

To follow up on the increased expression of several chemokines/cytokines detected in HD conditions, we tested the levels of chemokines/cytokines secreted from MCF7 cells and confirmed an increase in CXCL10, a key chemoattractant of cytotoxic T cells, NK cells, and Th1 cells (60), in HD conditions. Expression of the Y537S-ER completely suppressed the increased secretion of CXCL10 in HD condition, validating that this effect was through ER inhibition (Supplementary Fig. S5B). More broadly, the transcriptional differences between E2 and HD conditions after 24 hours of IFNγ stimulation were diminished in the presence of the Y537S mutation (Supplementary Fig. S5C and S5D). A direct comparison between WT versus Y537S mutant cells in HD conditions after IFNγ stimulation revealed increased expression of B2M, IRF1, STAT1, CXCL9, and CXCL10 in the WT-ER cells. Pathway analysis showed that the genes upregulated in WT-ER cells are involved in IFN response pathways, whereas the genes upregulated in Y537S-ER mutant cells are enriched in ER-related pathways (Supplementary Fig. S5E–S5G), further supporting the role of the inhibition of the ER transcriptional axis in the upregulation of IFN signaling.

To delineate the chromatin changes and identify the transcription factors involved in the disparate responses to IFNγ in HD and E2 conditions, we studied chromatin accessibility by applying the Assay for Transposase-Accessible Chromatin using sequencing (ATAC-seq). When we compared E2 versus HD conditions with and without IFNγ stimulation, we identified 549 sites with gained chromatin accessibility in the E2 conditions (Fig. 4I). As expected, these sites were enriched in ERE and FOXA1 motifs (Supplementary Fig. S5H). There was a limited number of accessible sites gained in HD conditions (*N* = 138). These were gained in HD conditions without and with IFNγ stimulation and enriched in TEAD and AP1 motifs with a trend toward enrichment of RelA (NF-κB) motifs (Supplementary Fig. S5I). Intriguingly and in keeping with the RNA-seq data, when we tested the accessible sites gained after IFNγ comparing HD and E2 conditions, we identified 2266 IFNγ-stimulated sites unique to HD, 946 sites shared to both conditions, and only 294 sites unique to E2 conditions (Fig. 4J). As expected, all three groups of accessible sites were enriched in IRF motifs. There was also a trend toward the enrichment of the RelA motif in the HD unique sites (Fig. 4K). In aggregate, our RNA-seq and ATAC-seq data indicate that ER^+^ breast cancer cells are poised for an enhanced response to IFNγ after ER blockade, and IFNγ stimulation leads to an enhanced downstream response in HD conditions. Based on the RNA-seq pathway analysis and ATAC-seq motif analysis, we hypothesized that these findings are due to enhanced NF-κB signaling, a signaling pathway involved in key physiologic processes, including activation of the immune system, cell proliferation, and apoptosis (61).

### ER blockade augments RelA phosphorylation and transcriptional activity

**Figure 5.**
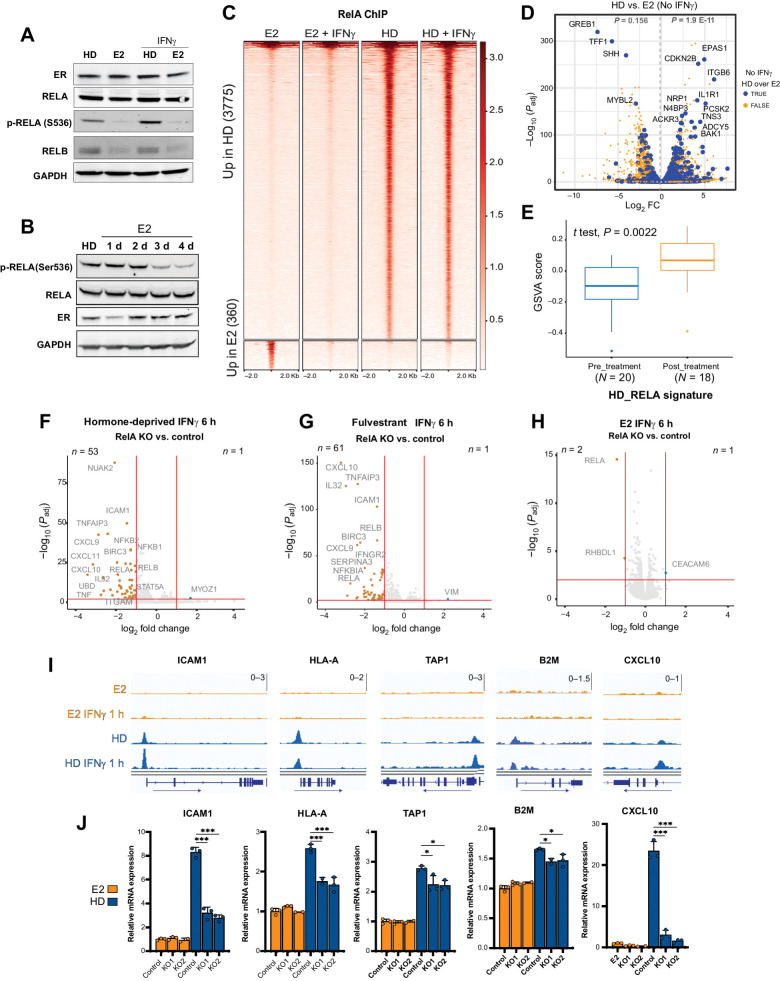
NF-κB pathway activation via RelA phosphorylation and binding is enhanced in hormone-deprived conditions. **A,** Immunoblot of whole-cell lysates for the NF-κB subunits, RelA and RelB, and ER in MCF7 cells grown with E2 10 nmol/L or in HD conditions in response to IFNγ 10 ng/mL stimulation. **B,** Whole-cell lysate immunoblots of ER, RelA, and phospho-RelA (Ser536) in MCF7 cells. Hormone-deprived cells were stimulated with E2 (10 nmol/L) for 4 days. Protein was extracted every 24 hours. GAPDH was used as a loading control. **C,** Tornado plots of RelA binding sites in HD cells or treated with 10 nmol/L E2 with or without IFNγ stimulation (10 ng/mL for 1 hour). **D,** Volcano plot showing differential expression from RNA-seq of MCF7 cells in HD conditions versus E2-treated conditions. Blue dots (True), genes that are differentially expressed based on RNA-seq and predicted to be regulated by RelA based on RelA ChIP-seq and BETA minus analysis. Orange dots (false), genes that are differentially expressed between HD and E2 conditions but not predicted to be regulated by RelA based on the RelA ChIP-seq data. The *P* value represents the significance of the association between RelA ChIP-seq and RNA-seq up HD (*P* = 1.9 E−11) or down in HD (*P* = 0.156) compared with E2-stimulated cells without IFNγ based on BETA basic. **E,** GSVA of the HD_RelA gene set (541 genes) in primary ER^+^ breast cancers pre- and post-neoadjuvant treatment with an AI. **F** and **G,** RNA-seq differential expression after RelA KO compared with control. Volcano plot highlighting genes differentially expressed between RelA KOs and RelA WT cells grown in HD conditions (**F**), treated with fulvestrant (10 nmol/L; **G**), or grown in E2 conditions (**H**) for 72 hours and stimulated with IFNγ (10 ng/mL) for the last 6 hours (h). *n*, number of genes differentially expressed. **I,** RelA ChIP-seq tracks showing examples of RelA peaks at the promoter region of IFNγ-associated genes in MCF7 cells grown in the presence of E2 or in HD conditions and stimulated ± IFNγ (10 ng/mL) for one hour. **J,** mRNA expression levels of ICAM1, HLA-A, TAP1, B2M, and CXCL10 in E2 and HD conditions without and with RELA silencing KO after 6 hours of IFNγ stimulation. *, *P* < 0.05; **, *P* < 0.01; ***, *P* < 0.001. Error bars, mean ± SD of at least two replicates per each KO. Two-way ANOVA.

To test our hypothesis that the enhanced transcription of IFNγ-related genes in HD condition in ER^+^ breast cancer cells is through NF-κB signaling, we first assessed the levels of RelA (p65), the subunit of the canonical NF-κB complex that contains the transcriptional transactivation domain (TAD). Although there were no significant changes in the level of total RelA in HD versus E2 conditions in MCF7 cells, phosphorylation of RelA at S536, a phosphorylation site within the TAD that promotes transactivation (62), was increased in HD conditions (Fig. 5A). In addition, RelB, a noncanonical subunit of NF-κB that also has a TAD and is a transcriptional target of RelA (63), was upregulated in HD conditions without and with IFNγ stimulation at the transcript and protein levels (Fig. 5A). Time-dependent decrease and increase in S536 phosphorylation (p-RelA) with E2 exposure and HD, respectively, further substantiated the role of HD in p-RelA (Fig. 5B; Supplementary Fig. S6A). This effect was most likely a direct consequence of ER inhibition and not an effect secondary to the cell-cycle arrest induced by E2 deprivation because treatment with the CDK4/6i palbociclib did not affect RelA phosphorylation or RelB expression (Supplementary Fig. S6B). This is also indicative of the distinct mechanisms by which palbociclib (55) and ET augment antigen presentation.

To test the effect of ER blockade on RelA-mediated transcription, we performed RelA ChIP-seq. Remarkably, RelA binding was primarily detected in HD conditions (3,775 RelA binding sites in HD conditions and only 360 binding sites in E2 conditions; Fig. 5C). As expected, the RelA (NF-κB) motif was the most significantly enriched motif in the RelA binding sites (Supplementary Fig. S6C). The AP-1 motif was the second most enriched motif, which is consistent with previous studies showing that NF-κB and AP-1 colocalize to form an inflammatory regulatory network (64). To test the direct transcriptional effects of RelA/NF-κB signaling in ER^+^ breast cancer in HD conditions, we integrated the RelA ChIP-seq (binding sites in HD conditions) and RNA-seq (differential expression between HD and E2 conditions), using the BETA algorithm (41). This analysis revealed a significant association between the RelA binding sites and the genes upregulated (but not downregulated) in HD conditions compared with E2 conditions without IFNγ stimulation (Fig. 5D). The BETA analysis also enabled us to identify the genes predicted to be direct RelA transcriptional targets in HD conditions (*N* = 541 genes, see Materials and Methods). These genes, defined as the HD_RelA gene set (Supplementary Table S11), are involved in pathways of NF-κB signaling and IFNγ response (Supplementary Fig. S6D). BETA analysis testing the genes that are direct targets of RelA in HD conditions after IFNγ treatment also showed a significant association between RelA binding after IFNγ treatment and genes upregulated in HD and IFNγ stimulated conditions, but not the genes upregulated in E2 conditions (Supplementary Fig. S6E; Supplementary Table S12). Notably, RelA binding was detected in the promoter region of IFNGR2 in HD conditions (Supplementary Fig. S6F), which is consistent with the RNA-seq data in which we detected the upregulation of IFNGR2 in HD conditions (Fig. 3H) and suggests that IFNGR2 is a direct transcriptional target of RelA. Moreover, RelA was the second top-ranked transcription factor predicted to regulate IFNGR2 in publicly available ChIP-seq data sets (Supplementary Fig. S6G; ref. 65). The clinical relevance of these findings is evidenced by the increased expression levels of the HD_RelA gene set after neoadjuvant treatment with an AI in primary ER^+^ breast cancer biopsies obtained pre- and posttreatment (Fig. 5E; ref. 46). In addition, the expression levels of the HD_RelA gene set inversely correlated with the ER levels in the TCGA cohort of primary ER^+^ breast cancers (Supplementary Fig. S6H).

To further validate that the transcriptional activity of RelA in ER^+^ breast cancer cells is nearly restricted to HD conditions, we silenced RelA with CRISPR-Cas9 using two different single guide RNAs (gRNA; Supplementary Fig. S6I and S6J). When assessing the transcriptional changes in a global manner, the effect of RelA silencing on transcription after 6 hours of IFNγ treatment was seen only in HD conditions (Fig. 5F) or after fulvestrant treatment (Fig. 5G), with nearly no differentially expressed genes apart from RelA itself in E2 conditions (Fig. 5H). In addition, RelA silencing resulted mainly in the downregulation of gene expression, with the upregulation of only one gene in all three conditions (HD, fulvestrant, E2). These results corroborated our ChIP-seq and RNA-seq data, which showed that RelA-mediated chromatin binding and transcription are dependent on ER blockade, and the direct transcriptional effect is predominantly gene upregulation.

Although we detected transcriptional effects upon RelA silencing in HD conditions, these effects were relatively limited because only 53 genes significantly downregulated after 6 hours of IFNγ treatment. This suggests that there may be other components of the NF-κB complex or other transcription factors that compensate for RelA loss. Nonetheless, RelA silencing resulted in the downregulation of IFNγ target genes with key roles in antigen presentation that we identified as direct RelA transcriptional targets in ER^+^ breast cancer cells, such as ICAM1 (66), HLA-A, TAP1, B2M, and CXCL10 (Fig. 5I and J). The impact of RelA silencing on B2M was statistically significant, but the absolute difference was limited, which is consistent with the notion that other transcription factors, such as IRF1, facilitate the upregulation of B2M in ER^+^ breast cancer cells as in other cell types (67). Conversely, RelA was found to be essential for CXCL10 expression, as RelA silencing led to near-complete loss of CXCL10 transcription.

### Birinapant potentiates the antitumoral effect of ER blockade and enhances tumor immunogenicity

We next sought to investigate whether the effect of ET on NF-KB can be leveraged for therapeutic purposes. To explore this, we examined the effects of combining a SMAC mimetic with ET in ER^+^ breast cancer models. SMAC mimetics are a class of proapoptotic agents currently under clinical development that induce cIAP/XIAP protein degradation, leading to enhanced apoptosis. Additionally, they promote NF-κB signaling by stabilizing NFκB-inducing kinase (NIK) expression (68). Importantly, NF-κB exerts prosurvival effects partly through the induction of the cIAP proteins that interact with TRAF2 (69). Therefore, SMAC mimetics have the potential to enhance the immune effects while blocking the prosurvival effects of NF-κB. Moreover, a recent study showed that the SMAC mimetic, birinapant, increased response to ICIs in *in vivo* syngeneic models of melanoma (70).

**Figure 6.**
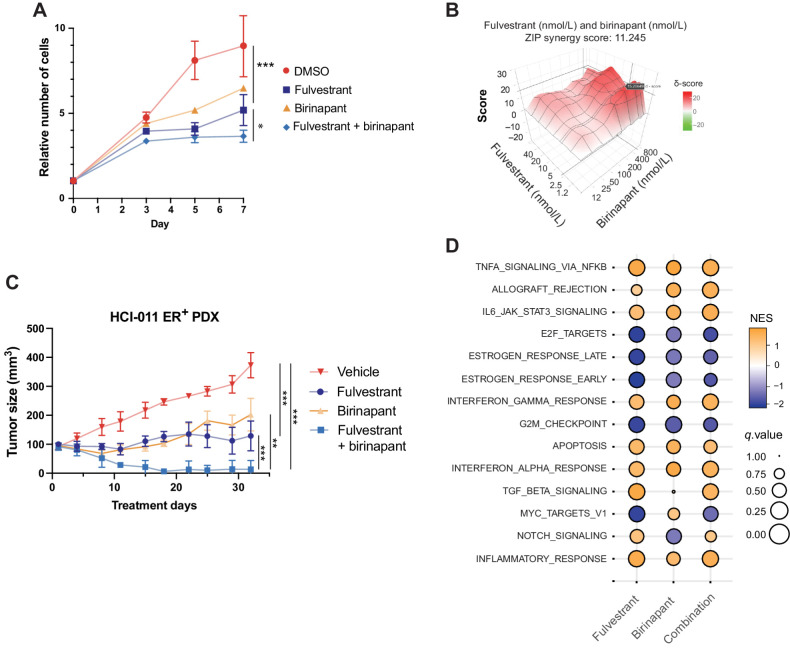
Birinapant potentiates the antitumoral effect of ER blockade. **A,** Cell growth studies of MCF7 cells treated with vehicle (DMSO), fulvestrant, birinapant, and the combination of fulvestrant and birinapant for 7 days. The number of cells were measured at days 0, 3, 5, and 7. Error bars, SD from four replicates. **B,** Synergy study of the combination of fulvestrant and birinapant MCF7 cells. Synergy was calculated based on the ZIP reference model using SynergyFinder (www.synergyfinder.org). Deviations between observed and expected responses with positive and negative values denote synergy and antagonism, respectively. **C,** ER^+^/HER2^−^ PDX tumor growth study. Error bars, SD (*N* = 5 mice per group). **D,** GSEA of genes differentially expressed in MCF7 cells after treatment with fulvestrant (10 nmol/L), birinapant (100 nmol/L), or the combination compared with vehicle (DMSO) control. The size of each circle indicates the *q* value (genes sets with a *q* value of <0.25 in at least one condition were included); the color scale indicates the normalized enrichment score (NES). *, *P* < 0.05; **, *P* < 0.01; ***, *P* < 0.001; two-way Anova.

We assessed the effect of birinapant on growth in ER^+^ breast cancer cells. Single-agent birinapant had a modest impact on cell growth as a single agent but enhanced the activity of fulvestrant (Fig. 6A). Remarkably, the combination of birinapant and fulvestrant was highly synergistic (Fig. 6B). Moreover, in an *in vivo* experiment of an ER^+^/HER2^−^ PDX, single-agent birinapant significantly decreased tumor growth and the combination of birinapant and fulvestrant was superior with complete tumor regression in 4 of the 5 mice (Fig. 6C; Supplementary Fig. S7A–S7D).

To investigate the mechanisms of the cell autonomous enhanced antitumor activity with the combination of birinapant and fulvestrant, we performed RNA-seq after 72 hours of treatment with vehicle, birinapant (100 nmol/L), fulvestrant (10 nmol/L), and the combination of these two drugs. GSEA of the transcriptomic changes after treatment with birinapant, fulvestrant, and the combination showed upregulation of the apoptosis pathway (Supplementary Table S13) and inhibition of ER signaling and the cell cycle (Fig. 6D). These convergent transcriptional effects may explain the synergy we observed. Pathway analysis also revealed an increase in NF-κB signaling and IFNγ and IFNα response with each of these drugs as single agents as well as the combination of these two drugs.

**Figure 7.**
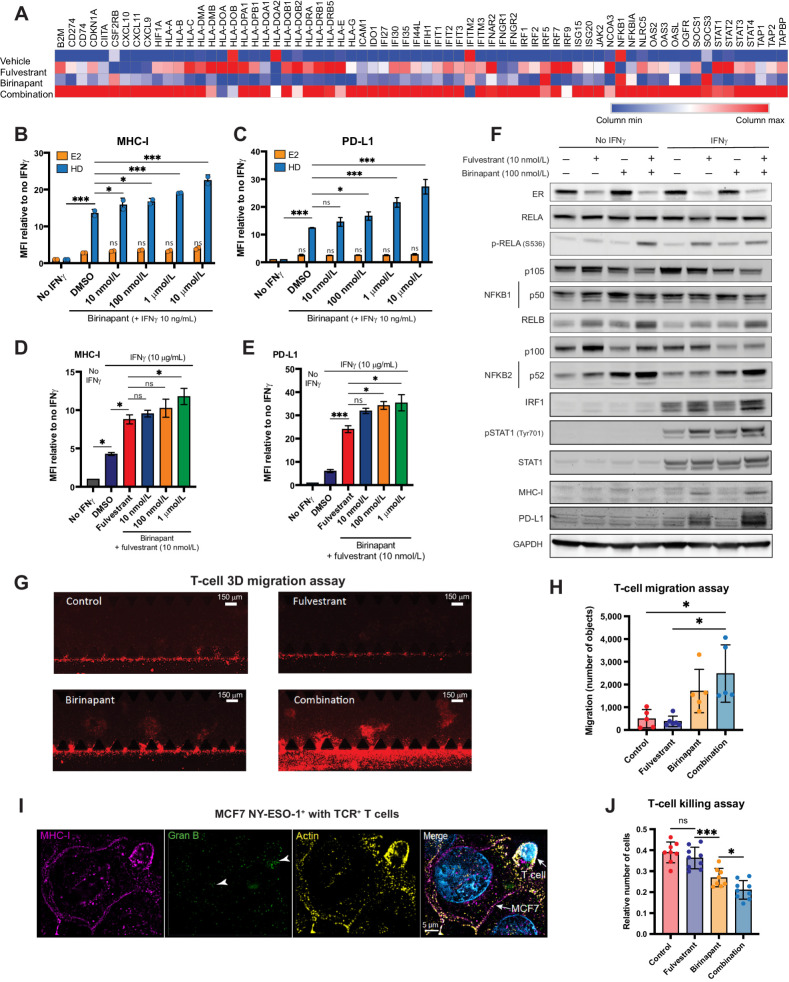
Birinapant and fulvestrant enhance antigen presentation, T-cell migration, and T-cell–mediated cytotoxicity. **A,** mRNA expression levels of IFNγ and antigen presentation–related genes in MCF7 cells that were treated with vehicle control (DMSO), fulvestrant (10 nmol/L), birinapant (100 nmol/L), and the combination of fulvestrant and birinapant for 3 days and stimulated with IFNγ for the last 24 hours. **B–E,** Median fluorescence intensity (MFI) quantification of MHC-I and PD-L1 levels assessed by flow cytometry in MCF7 cells grown in HD or in the presence of E2 (**B** and **C**) or treated with fulvestrant (10 nmol/L; **D** and **E**), and treated with the addition of doses of birinapant for 3 days. Cells were stimulated with IFNγ (10 ng/mL) for the last 24 hours. MFI levels are relative to no-IFNγ conditions (*, *P* < 0.05; ***, *P*< 0.001; n.s., not significant). Error bars, mean ± SD of at least two replicates. **F,** Whole-cell lysates immunoblots of ER, NF-KB subunits, and IFNγ target genes in MCF7 cells grown treated with vehicle control (DMSO), fulvestrant (10 nmol/L), birinapant (100 nmol/L), and the combination with and without IFNγ (10 ng/mL) stimulation for 24 hours. **G,** Representative immunofluorescence images from T-cell migration assay after treatment with vehicle control (DMSO), fulvestrant (10 nmol/L), birinapant (100 nmol/L), and the combination MCF7 cells were pretreated and seeded in the AIM 3D cell culture chips. Primary CD8^+^ T cells stained with CellTrace Red Stain were seeded on the lateral channels. **H,** Quantification of migration to the matrix was measured after 5 days (*, *P* < 0.05. Error bars are mean ± SD of at least 5 replicates. Two-way ANOVA). **I,** Representative figures of immunofluorescent stains of cocultured MCF7_NYESO1 and T cells transduced with NYESO1-specific TCR. Immunofluorescent stains include MHC-I (red), granzyme B (green), actin (yellow), DAPI for nuclear staining (blue), and a merged image. **J,** MCF7 cells expressing specific NYESO1 antigen were pretreated with vehicle (DMSO), fulvestrant (10 nmol/L), birinapant (100 nmol/L), and the combination of both drugs and cocultured with primary NYESO1 TCR^+^ T cells for 16 hours. The number of cancer cells alive was measured, and data are relative to MCF7_NYESO1 grown without T cells. *, *P* < 0.05; ***, *P* < 0.001; n.s., not significant. Error bars are mean ± SD of at least three replicates. Two-way ANOVA.

When comparing the genes induced by IFNγ in the presence of fulvestrant, birinapant, and the combination (log_2_ FC > 1, *q* < 0.01), we detected 293 shared genes and 167 genes that were uniquely upregulated with the combination (Supplementary Fig. S8A). Furthermore, K-means analysis identified three gene sets: (i) A gene set (defined as immune) that increased after IFNγ treatment with vehicle control and was further upregulated with the combination of fulvestrant and birinapant (*N* = 407 genes). This gene set was enriched in genes involved in IFNγ response, IFNα response, allograft rejection, complement, and TNFα signaling via NF-κB. (ii) A gene set we termed inflammatory (*N* = 219 genes) that increased after IFNγ stimulation in the presence of fulvestrant and the combination of fulvestrant and birinapant. (iii) The third gene set defined as ER (*N* = 317 genes) was downregulated after treatment with fulvestrant and the combination of fulvestrant and birinapant (Supplementary Fig. S8B). Looking at specific genes, we identified key members of the antigen-presenting machinery, including B2M, HLA-A/B, TAP1, TAP2, NLRC5, and the IFNγ–JAK–STAT pathway (*IFNG1*, *IFNG2*, and IRF1) among the genes that had enhanced expression with the combination of birinapant and fulvestrant compared with vehicle control or each drug alone (Fig. 7A).

At the protein level, birinapant enhanced MHC-I and PD-L1 expression in a dose-dependent manner in HD and IFNγ stimulated conditions but not in E2-treated conditions (Fig. 7B and C). Likewise, increasing concentrations of birinapant in combination with fulvestrant enhanced MHC-I and PD-L1 expression (Fig. 7D and E). Detailed protein analysis of the NF-κB complex showed that birinapant alone or in combination with fulvestrant did not affect RelA, p105, and p50 (NFκB1) levels (Fig. 7F). In contrast, birinapant in combination with fulvestrant increased RelA S536 phosphorylation prior to and after IFNγ stimulation. After IFNγ stimulation, the combination of birinapant and fulvestrant increased RelB levels. In addition, birinapant alone and in combination with fulvestrant increased the processing of the inactive p100 NFκB2 protein to p52, the active NFκB2 protein, prior to and after IFNγ stimulation, This is in keeping with the role of SMAC mimetics in stabilization of NIK and subsequent phosphorylation and activation of IKKα (Fig. 7F; ref. 68). Phosphorylation of STAT1 (pSTAT1) and IRF1, both downstream to *IFNG2*, a RelA transcriptional target gene, was detected only after IFNγ stimulation and had increased levels after treatment with fulvestrant or the combination of fulvestrant and birinapant (Fig. 7F). Similarly, in T47D cells, we observed an increase in p-RelA after fulvestrant treatment, which was enhanced with the combination of fulvestrant and birinapant (Supplementary Fig. S8C). Likewise, RelB, MHC-I, and IRF1 expression increased in T47D cells after treatment with the combination of fulvestrant and birinapant in IFNγ stimulated conditions (Supplementary Fig. S8C).

Profiling of the secretome (*N* = 105) in the culture medium of MCF7 cells treated with fulvestrant, birinapant, and the combination revealed that the combination of fulvestrant and birinapant augmented CXCL10 and CXCL9 secretion, supporting the RNA-seq results (Supplementary Fig. S8D and S8E). To determine the functional consequences of this finding, we performed a T-cell 3D migration test (Supplementary Fig. S8F) and detected increased T-cell migration toward MCF7 tumor spheroids in the presence of birinapant, which was significantly increased when fulvestrant was combined with birinapant (Fig. 7G and H). To test the effects of fulvestrant and birinapant on the antitumor activity of T cells mediated by specific recognition of an MHC-I–peptide complex by the specific TCR in ER^+^ breast cancer, we developed a coculturing assay. We stably expressed NY-ESO-1 in MCF7 cells, which have an HLA-A*0201 genotype, and generated CD8^+^ T cells stably expressing a TCR that specifically recognizes the complex of the NY-ESO-1 peptide antigen bound to MHC-I with an HLA-A*0201 allotype. After coculturing the NY-ESO-1-MCF7 cells and the activated engineered T cells, we observed increased T-cell mediated cytotoxicity with evidence of granzyme B secretion from the T cells after birinapant treatment and an enhanced effect with fulvestrant plus birinapant (Fig. 7I–J). In contrast, after coculturing NY-ESO-1-MCF7 cells with CD8^+^ T cells without the expression of the engineered TCR, there was no evidence of granzyme B secretion or T-cell–mediated cytotoxicity (Supplementary Fig. S8G–S8H), validating the role of specific antigen peptide–TCR interaction in the observed cytotoxicity. In aggregate, these results provide a 2-fold rationale for the combination of ET and birinapant, including (i) cell autonomous synergistic tumor regression and (ii) enhanced immune-mediated cancer cell cytotoxicity through increased migration of T cells, and tumor infiltration with cytotoxic T cells along with increased antigen presentation and T-cell recognition of ER^+^ cancer cells.

## Discussion

In clinical trials, the activity ICIs in HR^+^ breast cancer was shown to be limited. Furthermore, there is a paucity of information about the effects of ET on the TME and tumor immunogenicity. We performed a spatial proteomics analysis in primary HR^+^ breast cancers before and after ET alone and in combination with palbociclib to evaluate key immune-related proteins that are determinants of tumor immunogenicity. The most significant effects of ET alone and in combination with palbociclib were increases in STING and B2M in both immune and ICECs regions. Although the levels of STING and B2M remained higher in the immune regions compared with the ICECs. In addition, in the ICEC regions, there was an increase in several immune check points, such as TIM3, B7-H3, and CTLA4. Although the predictive or prognostic significance of these findings is unknown, these findings provide compelling evidence for the role of ET in shaping the TME in HR^+^ breast cancer. Moreover, our results indicate that ET may improve outcomes when combined with immune therapies in ER^+^ breast cancer. Furthermore, our results suggest that the CTLA4-inhibitor ipilimumab, or inhibitors of B7-H3 or TIM-3 that are currently in clinical development (71, 72), may be more effective in combination with ET compared with PD-1 or PD-L1 inhibitors in HR^+^ breast cancer.

We investigated the mechanism by which ET increases the expression of MHC-I in invasive cancer cells. Through these studies, we found that ER blockade is required for NF-κB signaling in ER^+^ breast cancer cells, which in turn mediates the expression of B2M in addition to key cytokines such as CXCL10. The activation of NF-κB also enhances the activation of the IFNGR2–JAK–STAT pathway, supporting the interaction between the NF-κB and IRF transcriptional networks (73). These results are consistent with and provide a mechanism to the inverse correlation between ER signaling and antigen presentation that was seen in the recent analysis of a clinical trial of chemotherapy in combination with pembrolizumab in metastatic HR^+^ breast cancer (13). These results are also in line with studies that demonstrated a cross-talk between ER and NF-κB. Previous studies showed that ER inhibits NF-κB–mediated transcription in reporter gene assays, and a direct interaction between ER and NF-κB was detected in models of recombinant ER (74). We performed a genome-wide study and demonstrated that endogenous RelA binding is diminished when ER is active in ER^+^ breast cancer cells. Furthermore, we delineated the direct transcriptional consequences of NF-κB transcriptional activity after ER inhibition and provide evidence for the clinical relevance of these findings. We also demonstrated that ER inhibition upregulated RelA phosphorylation at a transactivation site. ER-mediated suppression of RelA phosphorylation was reported previously and attributed, at least in part, to the upregulation of the long noncoding RNA LINC00472 induced by ER (75). However, other potential mechanisms may contribute to the effect of ER on RelA phosphorylation. Overall, the mechanism by which ER facilitates RelA phosphorylation and how RelA phosphorylation affects RelA chromatin binding in ER^+^ breast cancer cells warrant additional investigation.

We showed that after ER blockade, ER^+^ breast cancer cells are poised for an enhanced response to IFNγ through NF-κB signaling. We observed that key genes of the antigen presentation machinery and cytokines are upregulated after ET prior to IFNγ stimulation. Given these findings, we investigated if enhancing NF-κB signaling could elicit an increased antitumor immune response in ER^+^ breast cancer by testing the effects of the SMAC mimetic birinapant. Previous studies showed that NF-κB signaling plays a role in acquired resistance to ET, raising concerns about the therapeutic potential of enhancing NF-κB signaling (76). However, SMAC mimetics have dual effects; stabilization of NIK to promote NF-KB–mediated immune signaling and induction of cIAP/XIAP protein degradation, leading to enhanced apoptosis. Moreover, NF-κB's prosurvival effects are partly mediated through the induction of the cIAP proteins (69). Consequently, as we demonstrated, the addition of the SMAC mimetic birinapant to ET increased the expression of NF-κB immune target genes, leading to the migration of T cells toward ER^+^ breast cancer cells and MHC-I-specific T-cell–mediated cell death in ER^+^ breast cancer cells, while also increasing tumor regression in a cell autonomous manner. Taken together, these results coupled with the safety profile of SMAC mimetics (77, 78) provide a rationale for ET plus SMAC mimetics in combination with immunotherapy in HR^+^ breast cancer.

## Supplementary Material

Supplementary Table S1 Table S1

Supplementary Tables S2-13 Supplementary Tables S2-13

Supplementary Fig. S1 Supplementary Fig. S1. Comprehensive analysis of protein expression data in immune and invasive cancer epithelial regions from in various patient cohorts.

Supplementary Fig. S2 Supplementary Fig. S2. Comprehensive analysis of protein expression changes before and after endocrine treatment

Supplementary Fig. S3 Supplementary Fig. S3. Summary of protein expression changes after 24 weeks of endocrine treatment (ET) and/or Palbociclib (palbo) treatment.

Supplementary Fig. S4 Supplementary Fig. S4. Schematic of flow cytometry experiments and differential impact of IFNg stimulation on HR+ breast cancer cells.

Supplementary Fig. S5 Supplementary Fig. S5. Analysis of the impact of the ER axis on the response to IFNg stimulation in HR+ breast cancer cells.

Supplementary Fig. S6 Supplementary Fig. S6. Effect of hormone deprivation and NFKB pathway activation HR+ breast cancer cells.

Supplementary Fig. S7 Supplementary Fig. S7. Impact of treatment with fulvestrant, birinapant, and their combination on a PDX model of HR+ breast cancer.

Supplementary Fig. S8 Supplementary Fig. S8. Comprehensive analysis of fulvestrant and birinapant treatment in HR+ breast cancer cell.
