# Supplementary material for: Endocrine Therapy Synergizes with SMAC Mimetics to Potentiate Antigen Presentation and Tumor Regression in Hormone Receptor–Positive Breast Cancer
Source: Cancer Res. 2023 Jul 14;83(19):3284–304. doi: 10.1158/0008-5472.CAN-23-1711 (PMC10543960; doi:10.1158/0008-5472.CAN-23-1711)
Supplement: Supplementary Table S1 — Table S1 [file can-23-1711_supplementary_table_s1_suppst1.pdf]

Supplementary Table 1. Baseline patient clinicopathological characteristics in the Nanotsring Cohort and PELOPS treatment cohort

Clinical feature	Nanostring Cohort			PELOPS Treatment Cohort		
	Endocrine tx<br>N=32	Endocrine tx +Palbociclib<br>N=79	Overall<br>N=111	Endocrine tx<br>N=61	Endocrine tx +Palbociclib<br>N=128	Overall<br>N=189
Menopausal status:						
Pre-menopausal	13 (40.6%)	28 (35.4%)	41 (36.9%)	21 (34.4%)	48 (37.5%)	69(36.5%)
Post-menopausal	19 (59.4%)	51 (64.6)	70 (63.1%)	40 (65.6%)	80 (62.5%)	120(63.5%)
Histological subtype:						
Invasive Ductal	17 (53.1)	41 (51.9%)	58 (52.3%)	30 (49.2%)	62 (48.4%)	92 (48.7%)
Invasive Lobular	11 (34.4%)	34 (43.0%)	45 (40.5%)	25 (41.0%)	57 (44.5%)	82 (43.4%)
Mixed	4 (12.5%)	4 (5.1%)	8 (7.2%)	5 (8.2%)	9 (7.0%)	14 (7.4%)
Other	0 (0%)	0 (0%)	0 (0%)	1 (1.6%)	0 (0%)	1 (0.5%)
Clinical Stage:						
Stage I	0 (0%)	4 (5.1%)	4 (3.6%)	3 (4.9%)	12 (9.4%)	15 (7.9%)
Stage II	30 (93.8%)	63 (79.7%)	93 (83.8%)	54 (88.5%)	97 (75.8%)	151 (79.9%)
Stage III	2 (6.2%)	12 (15.2%)	14 (12.6%)	4 (6.6%)	19 (14.8%)	23 (12.2%)
T stage:						
T1	2(6.2%)	8 (10.1%)	10 (9.0%)	7 (11.4%)	12 (9.4%)	19 (10.0%)
T2	22 (68.8%)	47 (59.5%)	69 (62.2%)	36 (59.0%)	75 (58.6%)	111 (58.7%)
T3	8 (25.0%)	20 (25.3%)	28 (25.2%)	17 (27.9%)	35 (27.3%)	52 (27.5%)
T4	0 (0%)	4 (5.1%)	4 (3.6%)	0 (0%)	5 (3.9%)	5 (2.6%)
Unknown	0 (0%)	0 (0%)	0 (0%)	1 (1.6%)	1 (0.8%)	2 (1.1%)

N Stage:						
N0	19 (59.4%)	43 (54.4%)	62 (55.9%)	33 (54.1%)	66 (51.6%)	99 (52.4%)
N1	12 (37.5%)	34 (43.0%)	46 (41.4%)	26 (42.6%)	55 (43.0%)	81 (42.9%)
N2	0 (0%)	0 (0%)	0 (0%)	1 (1.6%)	2 (1.6%)	3 (1.6%)
N3	0 (0%)	0 (0%)	0 (0%)	0 (0%)	1 (0.8%)	1 (0.5%)
Unknown	1 (3.1%)	2 (2.5%)	3 (2.7%)	1 (1.6%)	4 (3.1%)	5 (2.6%)
Baseline TILs						
Mean (SD)	0.058 (0.061)	0.077 (0.13)	0.071 (0.12)	0.051 (0.052)	0.076 (0.12)	0.068 (0.11)
Median	0.05	0.05	0.05	0.05	0.05	0.05
[Min, Max]	[0.01, 0.3]	[0.01, 0.8]	[0.01, 0.8]	[0.01, 0.3]	[0.01, 0.8]	[0.01, 0.8]
[Q1, Q3]	[0.01, 0.05]	[0.01, 0.05]	[0.01, 0.05]	[0.01, 0.05]	[0.01, 0.1]	[0.01, 0.05]
Missing (%)	1 (3.1%)	5 (6.3%)	6 (5.4%)	8 (13%)	23 (18%)	31 (16%)
Baseline IHC %Ki-67						
Mean (SD)	14.2 (12.6)	13.8 (12)	13.9 (12.2)	15.1 (15.7)	11.3 (11.3)	12.6 (13)
Median	10.2	12.3	11.3	9.31	8.25	8.72
[Min, Max]	[0, 45.6]	[0.015, 59.2]	[0, 59.2]	[0.005, 65.3]	[0.005, 59.2]	[0.005, 65.3]
[Q1, Q3]	[3.42, 23.5]	[3.62, 20.6]	[3.59, 21.6]	[2.43, 24.2]	[2.65, 15.8]	[2.64, 19]
Missing (%)	0 (0%)	1 (1.3%)	1 (0.9%)	1 (1.6%)	6 (4.7%)	7 (3.7%)
