# Supplementary material for: Endocrine Therapy Synergizes with SMAC Mimetics to Potentiate Antigen Presentation and Tumor Regression in Hormone Receptor–Positive Breast Cancer
Source: Cancer Res. 2023 Jul 14;83(19):3284–304. doi: 10.1158/0008-5472.CAN-23-1711 (PMC10543960; doi:10.1158/0008-5472.CAN-23-1711)
Supplement: Supplementary Fig. S1 — Comprehensive analysis of protein expression data in immune and invasive cancer epithelial regions from in various patient cohorts. [file can-23-1711_supplementary_fig.s1_suppsf1.pdf]

Supplementary Fig. S1. (A) Principal components of expression data for immune region observations for the top 25 most variable proteins within immune region observations, colored by their spatial orientation to invasive cancer cells. N indicates number of patients included in the corresponding analysis. **(B)** Heatmap of Nanostring digital spatial proteomics expression data for the top 25 most variable proteins for all observations with rows and columns hierarchically clustered with complete linkage. **(C)** Volcano plots of log2 fold changes and adjusted p-values of tests for differential protein expression comparing immune versus invasive cancer epithelial regions at baseline. Horizontal dotted line denotes a 5% FDR threshold. **(D-F)** Volcano plots of log2 fold changes and adjusted p-values of tests for differential protein expression between immune versus invasive cancer epithelial regions in patient cohorts: **[D]** at 2 weeks for all patients that received 2 weeks of endocrine treatment (ET) alone (including pre-menopausal women) **[E],** at the time of surgery for patients given endocrine ET for 24 weeks and **[F],** at the time of surgery for patients that received ET with Palbociclib for 24 weeks. Horizontal dotted lines denote 5% FDR thresholds. **(G-H)** Trajectory plots of progesterone receptor (PR) **[G],** and Ki-67 **[H],** in post-menopausal patients treated with Tamoxifen during the window of opportunity part of the study. **(I-J)** Trajectory plots of PR **[i],** and Ki-67 **[J],** in patients treated with letrozole during the window of opportunity part of the study. Boxplots show median, 25th, and 75th percentiles as boxes, the minimum of the 75th percentile + 1.5 * IQR and the maximum observation as the upper whisker and the maximum of the 25th percentile - 1.5 * IQR and the minimum observation as the lower whisker. Any observations outside the whiskers are plotted as individual points. **(K-N)** Scatterplots with Pearson correlation coefficients between IHC log2(%Ki-67 and average DSP log2(Ki-67) expression per patient with fit of a simple linear regression model for measurements at baseline **[K]**, 2 weeks **[L]**, surgery **[M]**, and log2 fold changes from baseline to 2 weeks **[N]**. N indicates the number of patients included in the corresponding analysis.

Supp Fig. S1

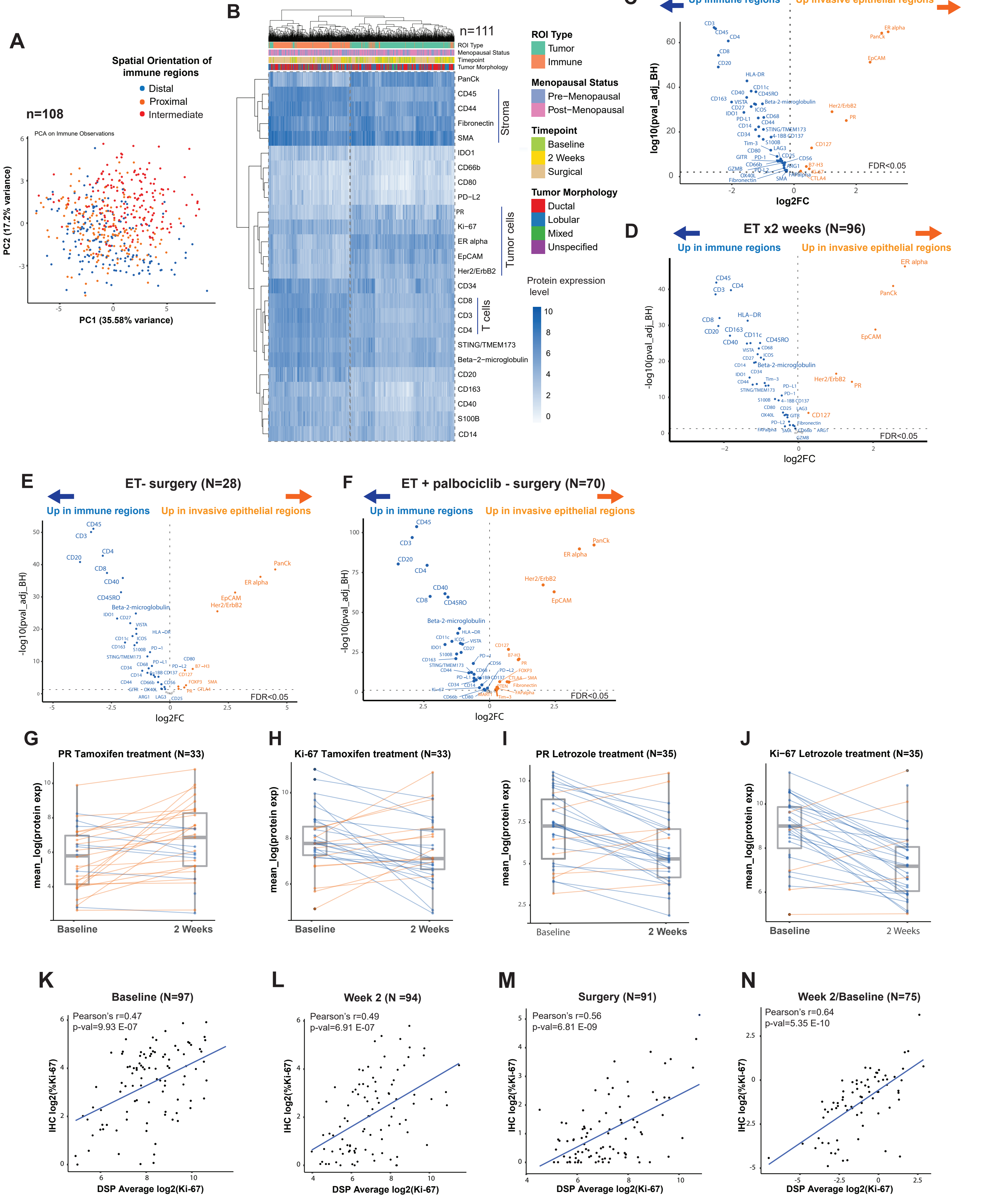
A
Spatial Orientation of immune regions
Distal
Proximal
Intermediate
n=108
PCA on Immune Observations
PC1 (35.58% variance)
PC2 (17.2% variance)
B
n=111
ROI Type
Tumor
Immune
Menopausal Status
Pre−Menopausal
Post−Menopausal
Timepoint
Baseline
2 Weeks
Surgical
Tumor Morphology
Ductal
Lobular
Mixed
Unspecified
Protein expression level
Stroma
Tumor cells
T cells
C
Baseline (n=102)
Up immune regions
Up invasive epithelial regions
log10(pval_adj_BH)
log2FC
FDR<0.05
D
ET x2 weeks (N=96)
Up in immune regions
Up in invasive epithelial regions
-log10(pval_adj_BH)
log2FC
FDR<0.05
E
ET- surgery (N=28)
Up in immune regions
Up in invasive epithelial regions
F
ET + palbociclib - surgery (N=70)
Up in immune regions
Up in invasive epithelial regions
G
PR Tamoxifen treatment (N=33)
H
Ki-67 Tamoxifen treatment (N=33)
I
PR Letrozole treatment (N=35)
J
Ki−67 Letrozole treatment (N=35)
mean_log(protein exp)
Baseline
2 Weeks
K
Baseline (N=97)
Pearson's r=0.47
p-val=9.93 E-07
L
Week 2 (N =94)
Pearson's r=0.49
p-val=6.91 E-07
M
Surgery (N=91)
Pearson's r=0.56
p-val=6.81 E-09
N
Week 2/Baseline (N=75)
Pearson's r=0.64
p-val=5.35 E-10
IHC log2(%Ki-67)
DSP Average log2(Ki-67)
