# Supplementary material for: Endocrine Therapy Synergizes with SMAC Mimetics to Potentiate Antigen Presentation and Tumor Regression in Hormone Receptor–Positive Breast Cancer
Source: Cancer Res. 2023 Jul 14;83(19):3284–304. doi: 10.1158/0008-5472.CAN-23-1711 (PMC10543960; doi:10.1158/0008-5472.CAN-23-1711)
Supplement: Supplementary Fig. S2 — Comprehensive analysis of protein expression changes before and after endocrine treatment [file can-23-1711_supplementary_fig.s2_suppsf2.pdf]

Supplementary Fig. S2. (A-B) Volcano plots of log2 fold changes and adjusted p-values of tests for differential protein expression comparing baseline versus after endocrine treatment (baseline versus surgery) in invasive epithelial regions **[A]** and immune regions **[B]**. Horizontal dotted lines denote 5% FDR thresholds. **(C-D)** Volcano plots comparing before pre and post 24 weeks (2 weeks versus surgical) of endocrine treatment (Tamoxifen (TAM) with Lupron (OS=ovarian suppression)) in pre-menopausal patients in the invasive epithelial cell regions [**C**] and immune regions **[D]. (E-F)** comparison of pre versus post endocrine treatment (Letrozole) (2 weeks versus surgical) in post-menopausal patients in the invasive epithelial cell regions **[E]** and immune regions **[F]**. (**G**) Correlation of differential mRNA and protein expression (log2 fold changes) comparing biopsies pre and post aromatase inhibitor treatment in NEOAI trial and PELOPS study (surgery versus 2 weeks including the immune regions)**. (H)** Correlation between the fold change of TILS fraction comparing surgery to baseline versus the fold change in Ki67 from 2-weeks to baseline including only patients that received letrozole in the window of opportunity part of the study and the treatment part **(I)** Correlation plot of the fold change of the immune related proteins with significant changes at the time of surgery over baseline and Ki67 fold change at 2 weeks over baseline only in patients who received letrozole treatment at the window of opportunity study and in the treatment part. N=9. * denotes p-value<0.05, Pearson's correlation. N indicates number of patients included in the corresponding analysis.

Supp Fig. S2

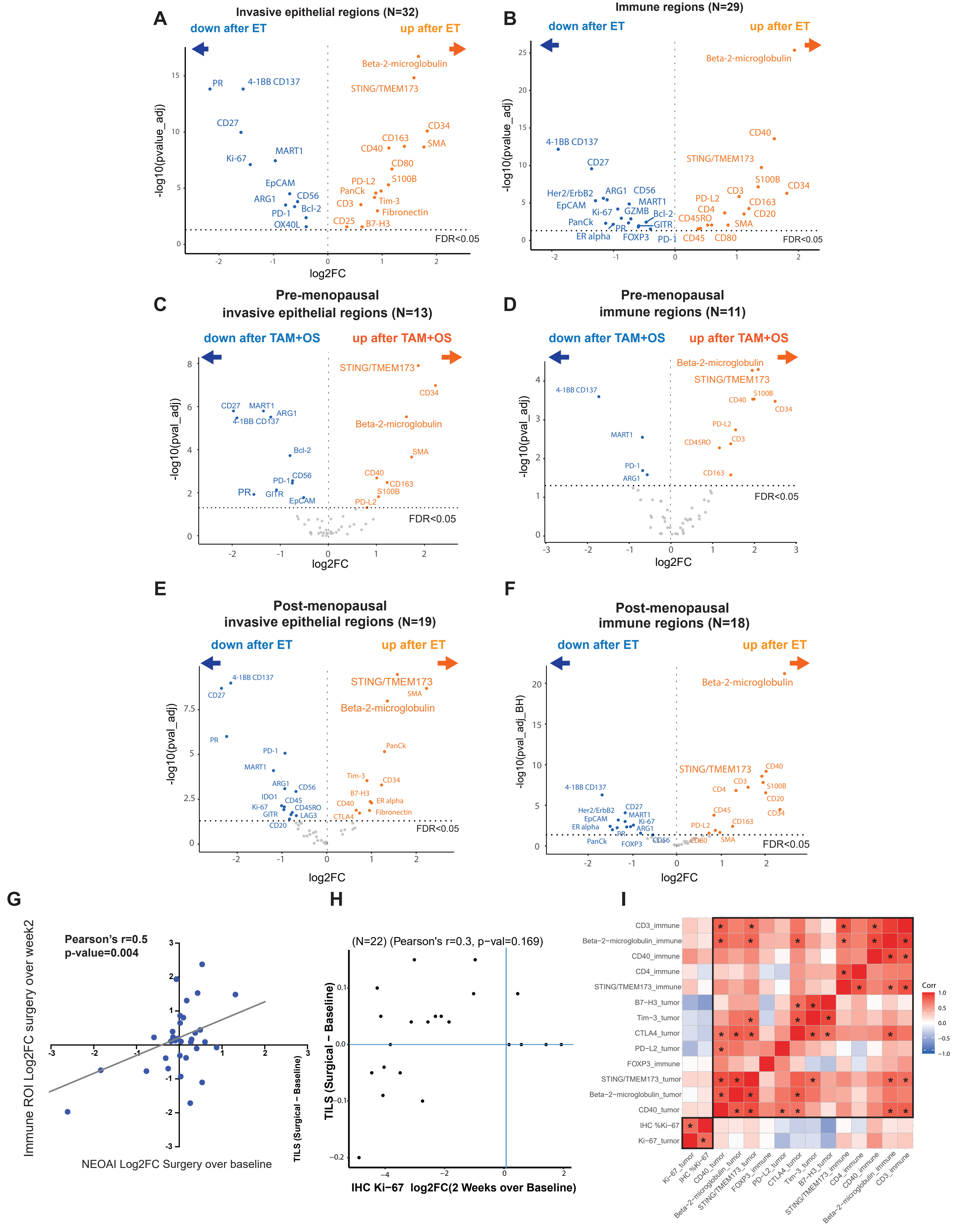

A
Invasive epithelial regions (N=32)
down after ET
up after ET
Beta-2-microglobulin
STING/TMEM173
PR
4-1BB CD137
CD27
CD34
CD163
SMA
CD40
MART1
Ki-67
CD80
EpCAM
PD-L2
S100B
PanCk
ARG1
CD56
Tim-3
Bcl-2
CD3
Fibronectin
PD-1
OX40L
CD25
B7-H3
FDR<0.05
-log10(pvalue_adj)
log2FC
B
Immune regions (N=29)
down after ET
up after ET
Beta-2-microglobulin
CD40
4-1BB CD137
STING/TMEM173
CD27
S100B
CD34
Her2/ErbB2
ARG1
CD56
CD3
EpCAM
MART1
PD-L2
CD163
Ki-67
GZMB
CD4
CD20
PanCk
Bcl-2
CD45RO
GITR
SMA
ER alpha
FOXP3
PD-1
CD45
CD80
FDR<0.05
-log10(pvalue_adj)
C
Pre-menopausal
invasive epithelial regions (N=13)
down after TAM+OS
up after TAM+OS
STING/TMEM173
CD34
CD27
MART1
ARG1
4-1BB CD137
Beta-2-microglobulin
Bcl-2
SMA
CD40
CD56
CD163
PD-1
PR
GITR
S100B
EpCAM
PD-L2
FDR<0.05
-log10(pval_adj)
log2FC
D
Pre-menopausal
immune regions (N=11)
down after TAM+OS
up after TAM+OS
Beta-2-microglobulin
STING/TMEM173
4-1BB CD137
S100B
CD40
CD34
PD-L2
MART1
CD3
CD45RO
PD-1
ARG1
CD163
FDR<0.05
-log10(pval_adj)
log2FC
E
Post-menopausal
invasive epithelial regions (N=19)
down after ET
up after ET
4-1BB CD137
CD27
STING/TMEM173
SMA
Beta-2-microglobulin
PR
PD-1
PanCk
MART1
Tim-3
CD34
ARG1
CD56
B7-H3
IDO1
CD45
ER alpha
Ki-67
CD45RO
CD40
GITR
LAG3
Fibronectin
CTLA4
CD20
FDR<0.05
-log10(pval_adj)
log2FC
F
Post-menopausal
immune regions (N=18)
down after ET
up after ET
Beta-2-microglobulin
STING/TMEM173
CD40
CD3
S100B
4-1BB CD137
CD4
CD20
CD27
Her2/ErbB2
CD45
CD34
MART1
EpCAM
Ki-67
ER alpha
ARG1
PD-L2
CD163
PR
PanCk
FOXP3
CD56
CD80
SMA
FDR<0.05
-log10(pval_adj_BH)
log2FC
G
Pearson's r=0.5
p-value=0.004
Immune ROI Log2FC surgery over week2
NEOAI Log2FC Surgery over baseline
TILS (Surgical – Baseline)
H
(N=22) (Pearson's r=0.3, p-val=0.169)
TILS (Surgical – Baseline)
IHC Ki-67 log2FC(2 Weeks over Baseline)
I
CD3_immune
Beta-2-microglobulin_immune
CD40_immune
CD4_immune
STING/TMEM173_immune
B7-H3_tumor
Tim-3_tumor
CTLA4_tumor
PD-L2_tumor
FOXP3_immune
STING/TMEM173_tumor
Beta-2-microglobulin_tumor
CD40_tumor
IHC %Ki-67
Ki-67_tumor
Corr
1.0
0.5
0.0
-0.5
-1.0
