# Supplementary material for: Endocrine Therapy Synergizes with SMAC Mimetics to Potentiate Antigen Presentation and Tumor Regression in Hormone Receptor–Positive Breast Cancer
Source: Cancer Res. 2023 Jul 14;83(19):3284–304. doi: 10.1158/0008-5472.CAN-23-1711 (PMC10543960; doi:10.1158/0008-5472.CAN-23-1711)
Supplement: Supplementary Fig. S3 — Summary of protein expression changes after 24 weeks of endocrine treatment (ET) and/or Palbociclib (palbo) treatment. [file can-23-1711_supplementary_fig.s3_suppsf3.pdf]

Supplementary Fig. S3. (**A-B)** volcano plot comparing pre versus post endocrine treatment (ET) plus Palbociclib (palbo) for 24 weeks (2 weeks versus surgical) in invasive epithelial regions **[A]** and immune regions **[B]**. Horizontal dotted lines denote 5% FDR thresholds. (**C-F**) Venn diagrams summarizing unique and shared differentially expressed proteins after 24 weeks of endocrine treatment (2 weeks versus surgical) and after Palbociclib plus endocrine treatment (ET) (2 weeks versus surgical) showing proteins up in invasive epithelial regions **[C]**, proteins up in immune regions **[D]**, proteins down in invasive epithelial regions **[e]**, and proteins down in immune regions **[F]**.

Supp Fig. S3

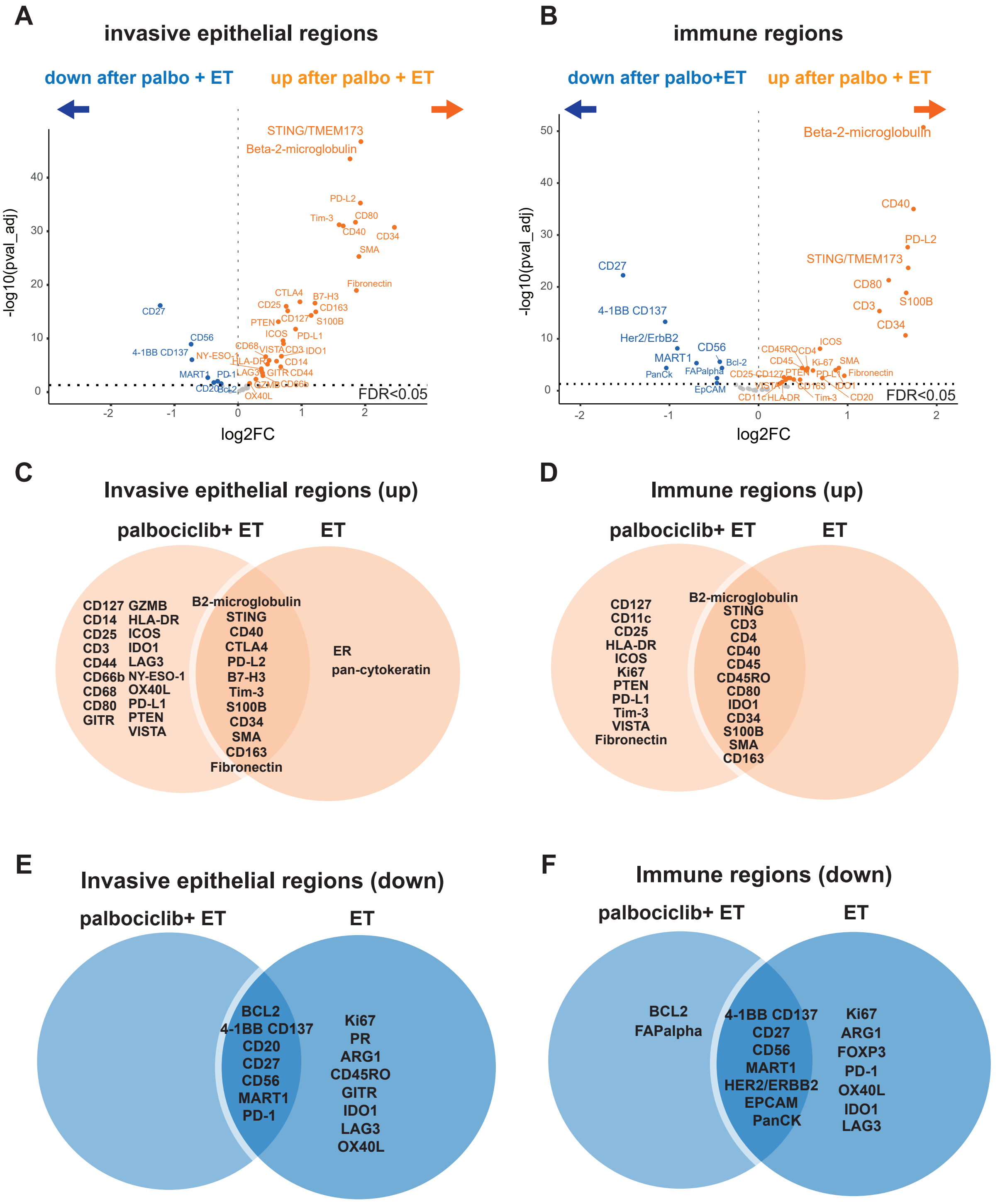
A
invasive epithelial regions
down after palbo + ET
up after palbo + ET
STING/TMEM173
Beta-2-microglobulin
PD-L2
Tim-3
CD80
CD40
CD34
SMA
Fibronectin
CTLA4
B7-H3
CD25
CD163
PTEN
CD127
S100B
ICOS
PD-L1
CD68
VISTA
CD3
IDO1
HLA-DR
CD14
LAG3
GITR
CD44
GZMB
CD66b
OX40L
CD27
CD56
4-1BB CD137
NY-ESO-1
MART1
PD-1
CD20
Bcl-2
FDR<0.05
-log10(pval_adj)
log2FC
B
immune regions
down after palbo+ET
up after palbo + ET
Beta-2-microglobulin
CD40
PD-L2
STING/TMEM173
CD80
S100B
CD3
CD34
CD27
4-1BB CD137
Her2/ErbB2
CD56
MART1
Bcl-2
PanCk
FAPalpha
EpCAM
CD45RO
CD4
ICOS
CD45
Ki-67
SMA
Fibronectin
CD25
CD127
PTEN
PD-L1
VISTA
CD163
IDO1
CD11c
HLA-DR
Tim-3
CD20
FDR<0.05
-log10(pval_adj)
log2FC
C
Invasive epithelial regions (up)
palbociclib+ ET
ET
CD127 GZMB
CD14 HLA-DR
CD25 ICOS
CD3 IDO1
CD44 LAG3
CD66b NY-ESO-1
CD68 OX40L
CD80 PD-L1
GITR PTEN
VISTA
B2-microglobulin
STING
CD40
CTLA4
PD-L2
B7-H3
Tim-3
S100B
CD34
SMA
CD163
Fibronectin
ER
pan-cytokeratin
D
Immune regions (up)
palbociclib+ ET
ET
CD127
CD11c
CD25
HLA-DR
ICOS
Ki67
PTEN
PD-L1
Tim-3
VISTA
Fibronectin
B2-microglobulin
STING
CD3
CD4
CD40
CD45
CD45RO
CD80
IDO1
CD34
S100B
SMA
CD163
E
Invasive epithelial regions (down)
palbociclib+ ET
ET
BCL2
4-1BB CD137
CD20
CD27
CD56
MART1
PD-1
Ki67
PR
ARG1
CD45RO
GITR
IDO1
LAG3
OX40L
F
Immune regions (down)
palbociclib+ ET
ET
BCL2
FAPalpha
4-1BB CD137
CD27
CD56
MART1
HER2/ERBB2
EPCAM
PanCK
Ki67
ARG1
FOXP3
PD-1
OX40L
IDO1
LAG3
