# Supplementary material for: Endocrine Therapy Synergizes with SMAC Mimetics to Potentiate Antigen Presentation and Tumor Regression in Hormone Receptor–Positive Breast Cancer
Source: Cancer Res. 2023 Jul 14;83(19):3284–304. doi: 10.1158/0008-5472.CAN-23-1711 (PMC10543960; doi:10.1158/0008-5472.CAN-23-1711)
Supplement: Supplementary Fig. S4 — Schematic of flow cytometry experiments and differential impact of IFNg stimulation on HR+ breast cancer cells. [file can-23-1711_supplementary_fig.s4_suppsf4.pdf]

Supplementary Fig. S4. (A) Schematic of the flow cytometry experiments. **(B)** Immunoblotting for MHC-I and PD-L1 in whole cell lysates of MCF7 cells that were grown for 3 days with E2 or HD conditions and stimulated with increasing concentrations of interferon-gamma (IFNg) for the last 24h, GAPDH was blotted as the loading control. **(C)** Histograms of MHC-I levels assessed by flow cytometry after 3 days of treatment with Fulvestrant and ARV-471 in the presence of IFNg for the last 24h in MCF7 cells expressing the Y537S ER mutation (ER-Y537S) under doxycycline treatment. **(D)** MFI quantification of **C)** (***denotes p<0.01, Two-way ANOVA). (**E**) Immunoblotting for STING in whole lysates of MCF7 and MDA-MB-231 (estrogen receptor negative) cells that were cultured in full media (FM), hormone deprived (HD) conditions and with estradiol (E2) for 3 days with or without IFNg (10ng/mL) stimulation. Histograms of PD-L1 levels following 3 days of E2 treated or HD conditions in the presence or absence of IFNg (10ug/mL) for the last 24h in MCF7 cells without doxycycline **(F)** or with doxycycline **(G)** induction of the ESR1 Y537S mutation (ND = no doxycycline, DOX = doxycycline). **(H)** Mean Fluorescence Intensity (MFI) quantification of the PD-L1 levels in **F**) and **G**) relative to no IFNg stimulation. (* denotes p< 0.05, NS is not significant. Two-way ANOVA). **(I)** Histograms of PD-L1 levels assessed by flow cytometry following 3 days of E2 treatment or HD conditions in the presence or absence of IFNg (10ug/mL) for the last 24h in MDA-MB-231 (ER negative) cells. **(J)** MFI quantification of PD-L1 levels compared to no IFNg stimulation from **I)** (n.s. denotes not significant, Two-way ANOVA)**.**

Supp Fig. S4

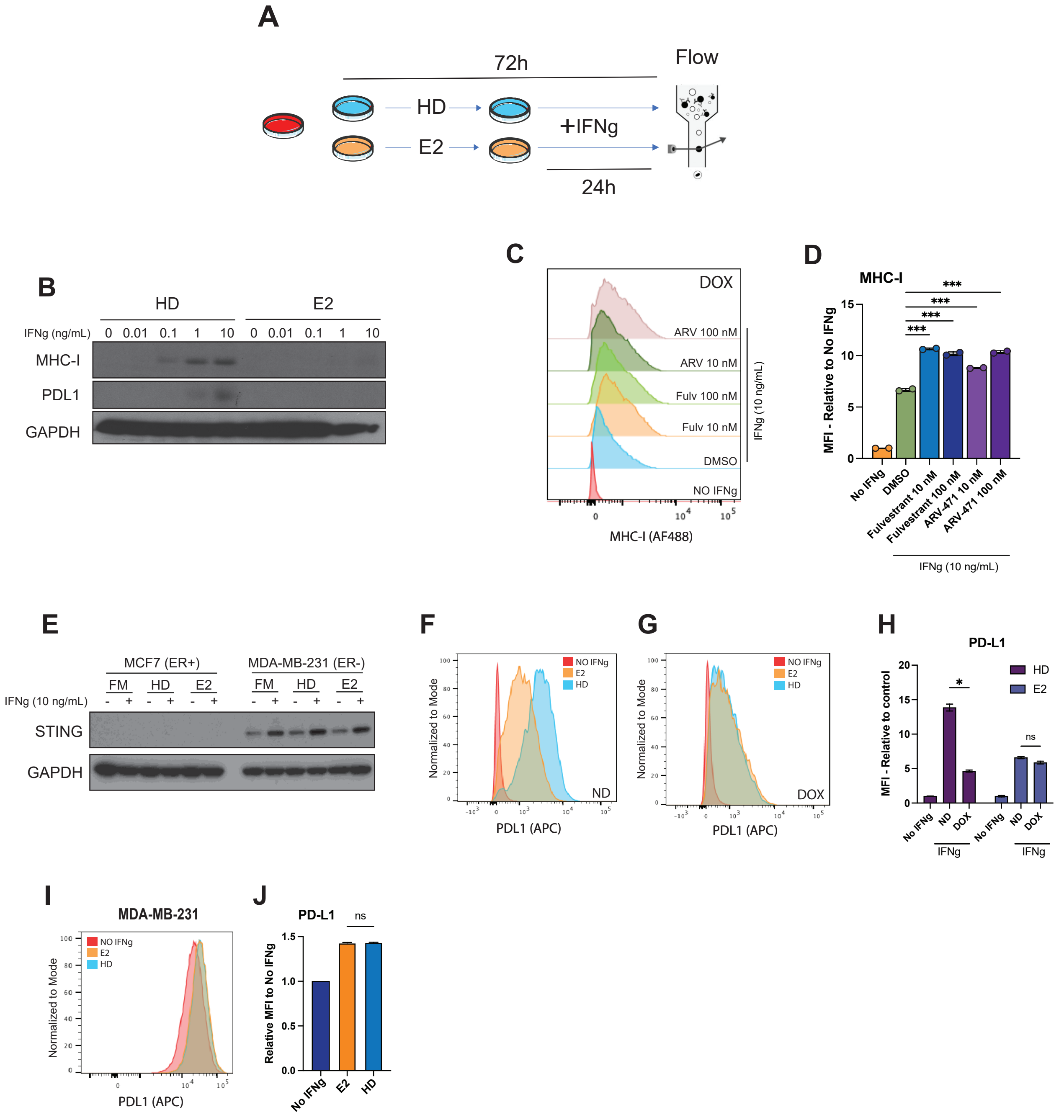
A
72h
Flow
HD
E2
+IFNg
24h
B
HD
E2
IFNg (ng/mL) 0 0.01 0.1 1 10 0 0.01 0.1 1 10
MHC-I
PDL1
GAPDH
C
DOX
ARV 100 nM
ARV 10 nM
Fulv 100 nM
Fulv 10 nM
DMSO
NO IFNg
IFNg (10 ng/mL)
MHC-I (AF488)
D
MHC-I
***
***
***
***
MFI - Relative to No IFNg
No IFNg
DMSO
Fulvestrant 10 nM
Fulvestrant 100 nM
ARV-471 10 nM
ARV-471 100 nM
IFNg (10 ng/mL)
E
MCF7 (ER+)
MDA-MB-231 (ER-)
FM HD E2 FM HD E2
IFNg (10 ng/mL) - + - + - + - + - + - +
STING
GAPDH
F
NO IFNg
E2
HD
Normalized to Mode
ND
PDL1 (APC)
G
NO IFNg
E2
HD
Normalized to Mode
DOX
PDL1 (APC)
H
PD-L1
HD
E2
*
ns
MFI - Relative to control
No IFNg
ND
DOX
No IFNg
ND
DOX
IFNg
IFNg
I
MDA-MB-231
NO IFNg
E2
HD
Normalized to Mode
PDL1 (APC)
J
PD-L1
ns
Relative MFI to No IFNg
No IFNg
E2
HD
