# Supplementary material for: Endocrine Therapy Synergizes with SMAC Mimetics to Potentiate Antigen Presentation and Tumor Regression in Hormone Receptor–Positive Breast Cancer
Source: Cancer Res. 2023 Jul 14;83(19):3284–304. doi: 10.1158/0008-5472.CAN-23-1711 (PMC10543960; doi:10.1158/0008-5472.CAN-23-1711)
Supplement: Supplementary Fig. S5 — Analysis of the impact of the ER axis on the response to IFNg stimulation in HR+ breast cancer cells. [file can-23-1711_supplementary_fig.s5_suppsf5.pdf]

Supplementary Fig. S5. (A) Number of genes induced by IFNg stimulation (10ng/mL) at 6h, 12h, 24 h and 24h in MCF7 cells treated with estradiol 10 nM (E2) or in hormone deprived (HD) conditions. DEseq2 Log2FC>1, adjusted p-value<0.01. **(B)** CXCL10 levels detected in the culture medium of MCF7 cells expressing wild-type ER (ER-WT) or expressing the ER Y537S mutation (ER-Y537S). Cells were cultured in HD or E2 conditions and stimulated with IFNg (10ng/mL) for 24h.*** denotes p-value< 0.001; NS denotes not significant. Two-way ANOVA. **(C-D)** Volcano plot of genes differentially expressed between HD and E2 treated conditions after IFNg (10ng/mL) stimulation for 24h in MCF7 cells expressing wild-type estrogen receptor (WT-ET) **[C]** or in MCF7 cells expressing the Y537S ER mutation (ER-Y537S) in a doxycycline inducible model **[D]**, DEseq2 Log2FC>1 and <-1, adjusted p-value<0.01. n indicates the number of genes significantly upregulated in each condition. **(E)** Volcano plot of genes differentially expressed between HD and E2 treated conditions after IFNg (10 ng/mL) stimulation for 24h in MCF7 cells with a Y537S ER mutation (ER-Y537S) knocked-in and expressed under the endogenous promoter, DEseq2 Log2FC>1 and <-1, adjusted p-value<0.01. n indicates the number of genes significantly upregulated in each condition. **(F-G)** Hallmark pathway analysis of genes upregulated in MCF7 cells expressing the Y537S ER mutation **[F]** or in MCF7 cells expressing the WT-ER **[G]** in HD conditions after IFNg stimulation (q-value< 0.05). **(H)** Motifs enriched in the chromatin accessible sites up in E2 treated conditions (Fig. 4i). **(I)** Motifs enriched in the chromatin accessible sites up in HD conditions (Fig. 4i).

Supp Fig. S5

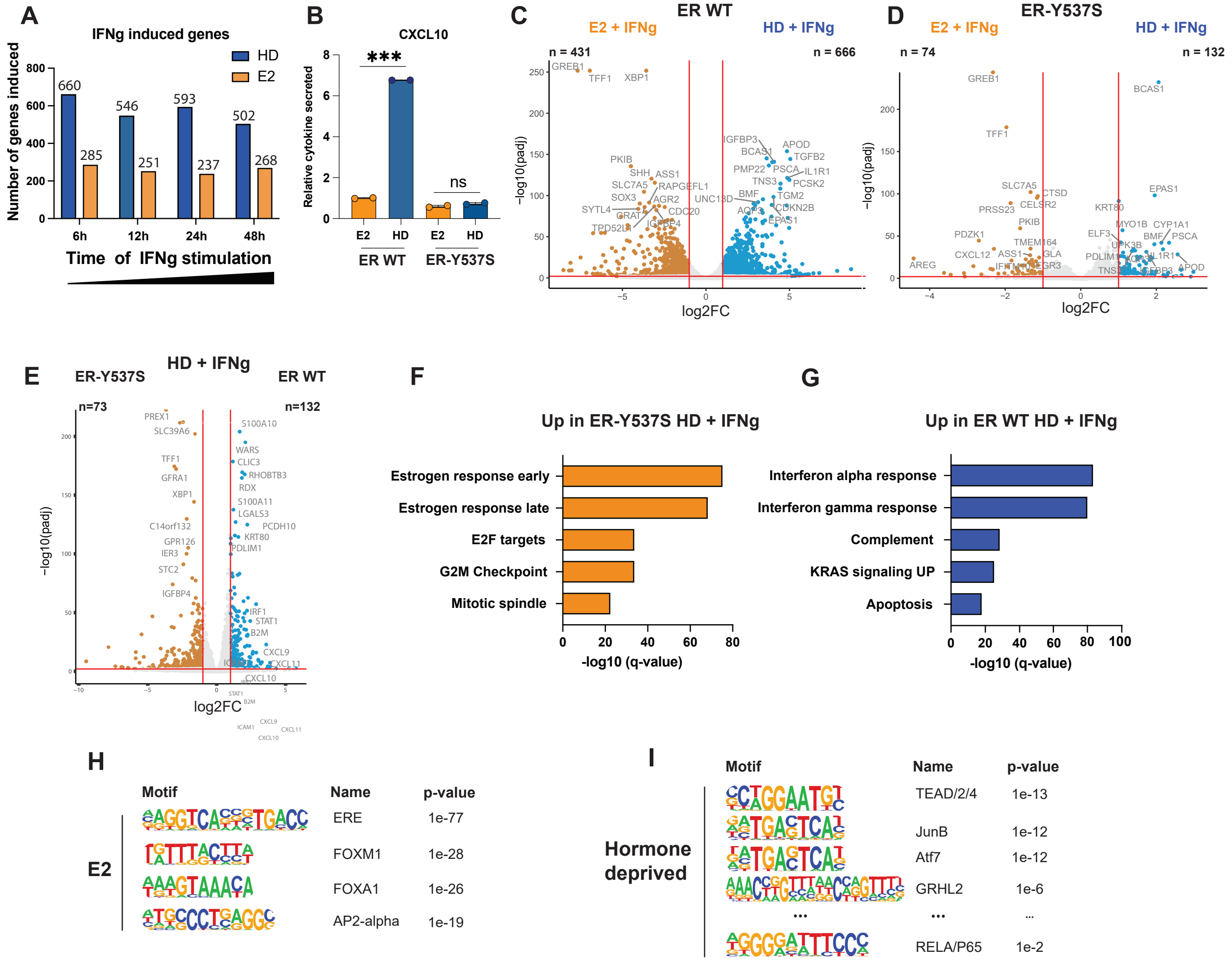
A
IFNg induced genes
HD
E2
Number of genes induced
660
285
546
251
593
237
502
268
6h
12h
24h
48h
Time of IFNg stimulation
B
CXCL10
Relative cytokine secreted
***
ns
E2
HD
E2
HD
ER WT
ER-Y537S
C
ER WT
E2 + IFNg
HD + IFNg
n = 431
n = 666
−log10(padj)
log2FC
D
ER-Y537S
E2 + IFNg
HD + IFNg
n = 74
n = 132
−log10(padj)
log2FC
E
HD + IFNg
ER-Y537S
ER WT
n=73
n=132
−log10(padj)
log2FC
F
Up in ER-Y537S HD + IFNg
Estrogen response early
Estrogen response late
E2F targets
G2M Checkpoint
Mitotic spindle
-log10 (q-value)
G
Up in ER WT HD + IFNg
Interferon alpha response
Interferon gamma response
Complement
KRAS signaling UP
Apoptosis
-log10 (q-value)
H
Motif
Name
p-value
E2
ERE
1e-77
FOXM1
1e-28
FOXA1
1e-26
AP2-alpha
1e-19
I
Motif
Name
p-value
Hormone deprived
TEAD/2/4
1e-13
JunB
1e-12
Atf7
1e-12
GRHL2
1e-6
RELA/P65
1e-2
