# Supplementary material for: Endocrine Therapy Synergizes with SMAC Mimetics to Potentiate Antigen Presentation and Tumor Regression in Hormone Receptor–Positive Breast Cancer
Source: Cancer Res. 2023 Jul 14;83(19):3284–304. doi: 10.1158/0008-5472.CAN-23-1711 (PMC10543960; doi:10.1158/0008-5472.CAN-23-1711)
Supplement: Supplementary Fig. S6 — Effect of hormone deprivation and NFKB pathway activation HR+ breast cancer cells. [file can-23-1711_supplementary_fig.s6_suppsf6.pdf]

Supplementary Fig. S6. (A) Immunoblot for RelA and phospho-RELA (Ser536) in MCF7 cells that were hormone deprived (HD) or treated with estradiol (E2), whole-cell lysates were extracted after 1, 2 or 3 days of hormone deprivation or E2 stimulation. GAPDH was used as loading control. **(B)** Immunoblot of the NFKB2 subunits (p52, p105), RelB, RelA and phospho-RelA of MCF7 cells treated with vehicle (DMSO) and increasing doses of Palbociclib for 72 hours. **(C)** Motif analysis of RelA ChIP-seq without interferon IFNg stimulation. **(D)** Volcano plot showing differential expression from RNA-seq of MCF7 cells in HD deprived conditions versus E2 treated conditions in the presence of interferon-gamma (IFNg). Blue dots denote genes that are differentially expressed based on RNA-seq and predicted to be regulated by RelA based on RelA ChIP-seq and BETA minus analysis (True). The orange dots (False) represent the genes that are differentially expressed between HD and E2 conditions but not predicted to be regulated by RelA based on the RelA ChIP-seq data. The p-value represents the significance of the association between RelA ChIP-seq and RNA-seq up HD (p-value=1.3 E-19) or down in HD (p-value = 0.66) compared to E2 stimulated cells with IFNg based on BETA basic. **(E)** **(F)** Hallmark Pathway analysis of the genes upregulated by RelA binding based on integration of RNA-seq and RelA ChIP-seq. **(g)** RelA binding peak at the transcription start site of *IFNGR2*. **(G)** Transcription factors that are the top ranked potential regulators of *IFNGR2*. **(H)** Gene set variation analysis (GSVA) of the HD_RelA gene set (541 genes) in the ER positive tumors from the TCGA cohort (N=802) divided to four quartiles based on the ESR1 mRNA levels. The comparison between the quartiles was done with a t-test. **(I)** Relative mRNA expression of RELA based on RNA-seq in MCF7 cells infected with CRISPR-Cas9 and a control gRNA or two different single gRNAs targeting *RELA*. **(J)** Immunoblot for RelA expression in MCF7 cells transduced with a control gRNA and with two different gRNAs targeting *RELA*.

Supp Fig. S6

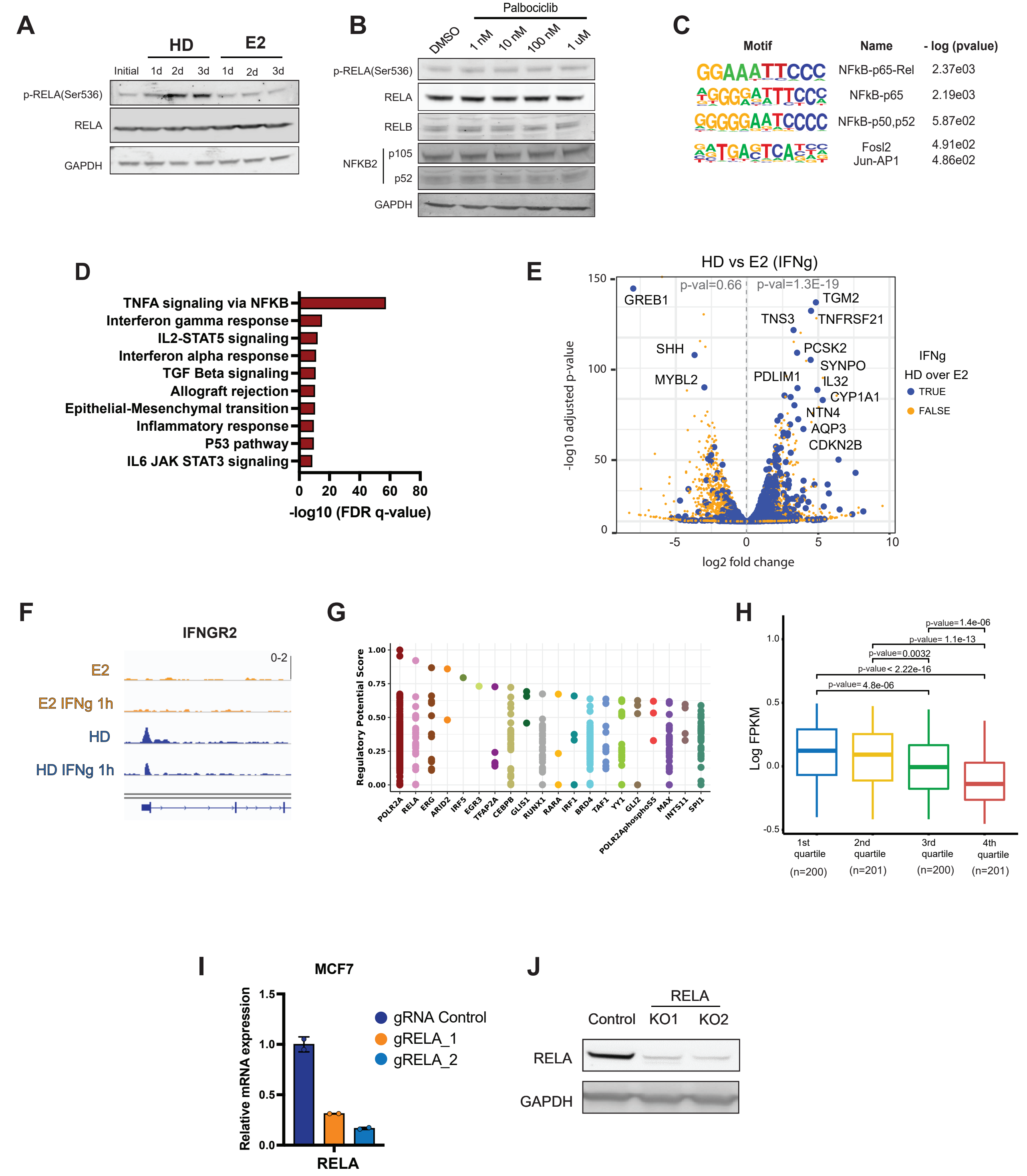
A
HD
E2
Initial
1d
2d
3d
1d
2d
3d
p-RELA(Ser536)
RELA
GAPDH
B
Palbociclib
DMSO
1 nM
10 nM
100 nM
1 uM
p-RELA(Ser536)
RELA
RELB
NFKB2
p105
p52
GAPDH
C
Motif
Name
- log (pvalue)
NFkB-p65-Rel
2.37e03
NFkB-p65
2.19e03
NFkB-p50,p52
5.87e02
Fosl2
Jun-AP1
4.91e02
4.86e02
D
TNFA signaling via NFKB
Interferon gamma response
IL2-STAT5 signaling
Interferon alpha response
TGF Beta signaling
Allograft rejection
Epithelial-Mesenchymal transition
Inflammatory response
P53 pathway
IL6 JAK STAT3 signaling
0
20
40
60
80
-log10 (FDR q-value)
E
HD vs E2 (IFNg)
p-val=0.66
p-val=1.3E-19
GREB1
TGM2
TNS3
TNFRSF21
SHH
PCSK2
SYNPO
PDLIM1
IL32
MYBL2
CYP1A1
NTN4
AQP3
CDKN2B
-log10 adjusted p-value
150
100
50
0
-5
0
5
10
log2 fold change
IFNg
HD over E2
TRUE
FALSE
F
IFNGR2
0-2
E2
E2 IFNg 1h
HD
HD IFNg 1h
G
Regulatory Potential Score
1.00
0.75
0.50
0.25
0.00
POLR2A
RELA
ERG
ARID2
IRF5
EGR3
TFAP2A
CEBPB
GLIS1
RUNX1
RARA
IRF1
BRD4
TAF1
YY1
GLI2
POLR2AphosphoS5
MAX
INTS11
SPI1
H
p-value=1.4e-06
p-value= 1.1e-13
p-value=0.0032
p-value< 2.22e-16
p-value= 4.8e-06
Log FPKM
1.0
0.5
0.0
-0.5
1st quartile
2nd quartile
3rd quartile
4th quartile
(n=200)
(n=201)
(n=200)
(n=201)
I
MCF7
Relative mRNA expression
1.5
1.0
0.5
0.0
RELA
gRNA Control
gRELA_1
gRELA_2
J
RELA
Control
KO1
KO2
RELA
GAPDH
