# Supplementary material for: Endocrine Therapy Synergizes with SMAC Mimetics to Potentiate Antigen Presentation and Tumor Regression in Hormone Receptor–Positive Breast Cancer
Source: Cancer Res. 2023 Jul 14;83(19):3284–304. doi: 10.1158/0008-5472.CAN-23-1711 (PMC10543960; doi:10.1158/0008-5472.CAN-23-1711)
Supplement: Supplementary Fig. S7 — Impact of treatment with fulvestrant, birinapant, and their combination on a PDX model of HR+ breast cancer. [file can-23-1711_supplementary_fig.s7_suppsf7.pdf]

Supplementary Fig. S7. (A-C) Individual PDX tumor size measured at different time points of vehicle treated group in comparison with fulvestrant (200 mg/kg) **[A]**, birinapant (20 mg/kg) **[B]** and the combination of fulvestrant (200 mg/kg) and birinapant (20 mg/kg) group **[C]**. **(D)** Pictures of PDX tumors at the end of the experiment.

Supp Fig. S7

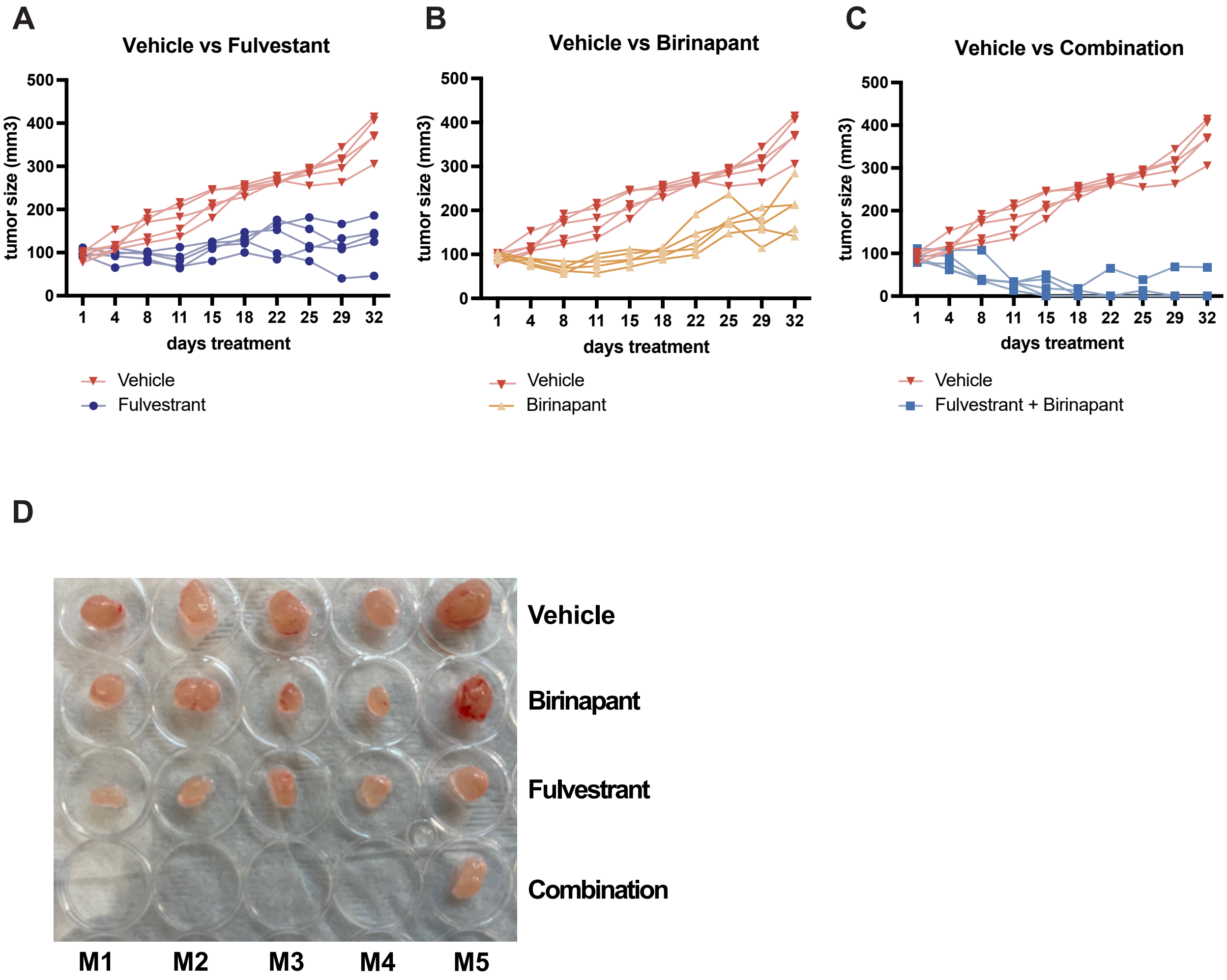

A
Vehicle vs Fulvestant
tumor size (mm3)
500
400
300
200
100
0
1
4
8
11
15
18
22
25
29
32
days treatment
Vehicle
Fulvestrant
B
Vehicle vs Birinapant
tumor size (mm3)
500
400
300
200
100
0
1
4
8
11
15
18
22
25
29
32
days treatment
Vehicle
Birinapant
C
Vehicle vs Combination
tumor size (mm3)
500
400
300
200
100
0
1
4
8
11
15
18
22
25
29
32
days treatment
Vehicle
Fulvestrant + Birinapant
D
Vehicle
Birinapant
Fulvestrant
Combination
M1
M2
M3
M4
M5
