# Supplementary material for: Endocrine Therapy Synergizes with SMAC Mimetics to Potentiate Antigen Presentation and Tumor Regression in Hormone Receptor–Positive Breast Cancer
Source: Cancer Res. 2023 Jul 14;83(19):3284–304. doi: 10.1158/0008-5472.CAN-23-1711 (PMC10543960; doi:10.1158/0008-5472.CAN-23-1711)
Supplement: Supplementary Fig. S8 — Comprehensive analysis of fulvestrant and birinapant treatment in HR+ breast cancer cell. [file can-23-1711_supplementary_fig.s8_suppsf8.pdf]

Supplementary Fig. S8. (A) Venn diagram depicting the number of overlapping or unique genes that are upregulated after interferon-gamma (IFNg 10ng/mL) treatment for last 24h during treatment with vehicle control (DMSO), fulvestrant (10 nM), birinapant (100 nM) or the combination for 72 hrs. **(B)** Unsupervised heatmap of RNA-seq from MCF7 in full medium (FM) cells treated with control, IFNg alone, IFNg in combination with birinapant (100 nM), fulvestrant (10 nM) or the combination. **(C)** Immunoblot of ER, phospho-RelA (Ser536), RelA, RelB, MHC-I (HLA-A, B, C) and IRF1 in T47D whole cell lysates treated with vehicle control (DMSO), fulvestrant (10 nM), birinapant (100 nM) or the combination for 72h and stimulated with IFNg (10 ng/mL) for the last 24 hours. **(D)** Human cytokine and chemokine levels in the culture medium of MCF-7 cells using a membrane-based antibody array. Cells were pretreated with vehicle (DMSO), fulvestrant (10 nM), birinapant (100 nM) or the combination for 48h. Cells were washed twice with PBS and treatments refreshed adding IFNg (10ng/mL) for 24h, without FBS to avoid detection of serum cytokines. **(E)** Quantification of signal from the membrane in [**F**] normalized to DMSO intensity. *** denotes a p-value <0.01, error bars are +/- SD, two-way ANOVA. **(F)** Schematic of the 3D T-cell migration assay. **(G)** Representative figures of immunofluorescent stains of MCF7_NYESO1 cells co-cultured with T- cells not transduced with NYESO1 specific TCR. Immunofluorescent (IF) stains include MHC-I (red) Granzyme B (green), Actin (yellow), DAPI for nuclear staining(blue) and a merge image. Of note, cells without expression of the NYESO1 specific TCR did not attach to the plate and are not detected after IF staining. **(H)** MCF7 cells expressing the NYESO1 antigen were pretreated with vehicle (DMSO), fulvestrant (10 nM), birinapant (100 nM) and the combination of both drugs and co-cultured with primary T cells not expressing the NYESO1 specific TCR for 16 hours. Number of cancer cells alive were measured and data is relative to MCF7_NYESO1 grown without T cells (Error bars are mean ± s.d of at least 3 replicates. Two-way ANOVA test showed no significant effects).

Supp Fig. S8

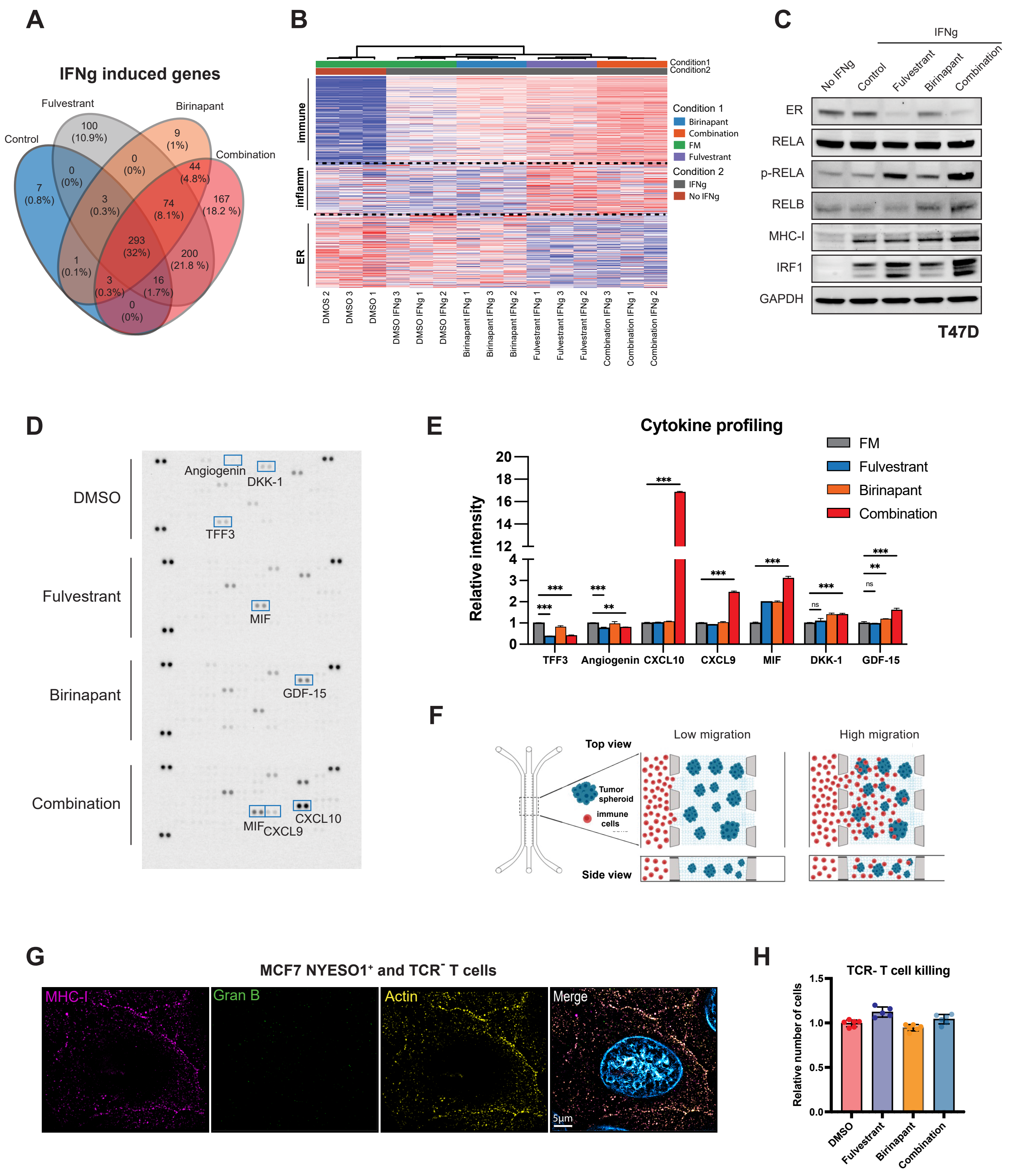
A
IFNg induced genes
Fulvestrant
Birinapant
Control
Combination
100
(10.9%)
9
(1%)
0
(0%)
7
(0.8%)
0
(0%)
44
(4.8%)
3
(0.3%)
74
(8.1%)
167
(18.2 %)
293
(32%)
1
(0.1%)
200
(21.8 %)
3
(0.3%)
16
(1.7%)
0
(0%)
B
Condition1
Condition2
immune
inflamm
ER
Condition 1
Birinapant
Combination
FM
Fulvestrant
Condition 2
IFNg
No IFNg
DMOS 2
DMSO 3
DMSO 1
DMSO IFNg 3
DMSO IFNg 1
DMSO IFNg 2
Birinapant IFNg 1
Birinapant IFNg 3
Birinapant IFNg 2
Fulvestrant IFNg 1
Fulvestrant IFNg 3
Fulvestrant IFNg 2
Combination IFNg 3
Combination IFNg 1
Combination IFNg 2
C
IFNg
No IFNg
Control
Fulvestrant
Birinapant
Combination
ER
RELA
p-RELA
RELB
MHC-I
IRF1
GAPDH
T47D
D
DMSO
Fulvestrant
Birinapant
Combination
Angiogenin
DKK-1
TFF3
MIF
GDF-15
MIF
CXCL9
CXCL10
E
Cytokine profiling
Relative intensity
FM
Fulvestrant
Birinapant
Combination
TFF3
Angiogenin
CXCL10
CXCL9
MIF
DKK-1
GDF-15
F
Top view
Low migration
High migration
Tumor spheroid
immune cells
Side view
G
MCF7 NYESO1+ and TCR- T cells
MHC-I
Gran B
Actin
Merge
5µm
H
TCR- T cell killing
Relative number of cells
DMSO
Fulvestrant
Birinapant
Combination
